# Supplementary material for: Molecular insights into capsular polysaccharide secretion
Source: Nature. 2024 Apr 3;628(8009):901–9. doi: 10.1038/s41586-024-07248-9 (PMC11041684; doi:10.1038/s41586-024-07248-9)
Supplement: Supplementary file 1 — Supplementary Figs 1–24 and Supplementary Table 1. [file 41586_2024_7248_MOESM1_ESM.pdf]

nature portfolio

https://doi.org/10.1038/s41586-024-07248-9

Supplementary information

Molecular insights into capsular polysaccharide secretion

In the format provided by the authors and unedited

SUPPLEMENTARY INFORMATION FOR

Molecular insights into capsular polysaccharide secretion

Jeremi Kuklewicz[1] and Jochen Zimmer[1,2*]

Affiliations

[1]Department of Molecular Physiology and Biological Physics, University of Virginia School of Medicine, Charlottesville, Virginia, USA

[2]Howard Hughes Medical Institute

*Corresponding author: jz3x@virginia.edu

Supplementary Information Figure 1a

Extended Data Fig. 1b

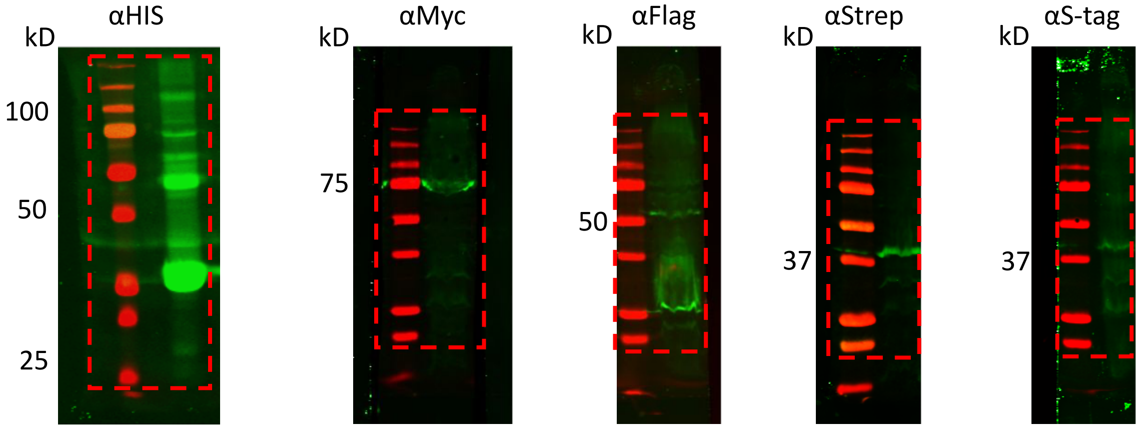

Extended Data Fig. 1c

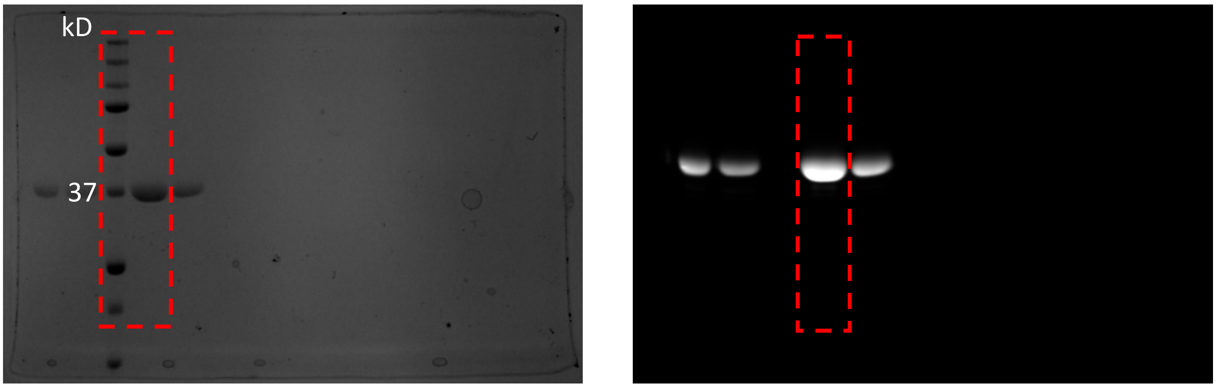

Extended Data Fig. 2b

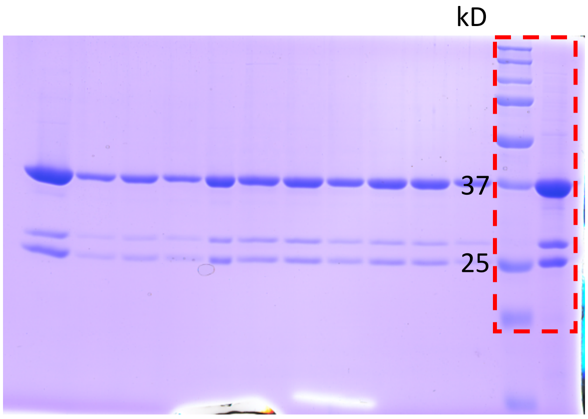

Extended Data Fig. 7a

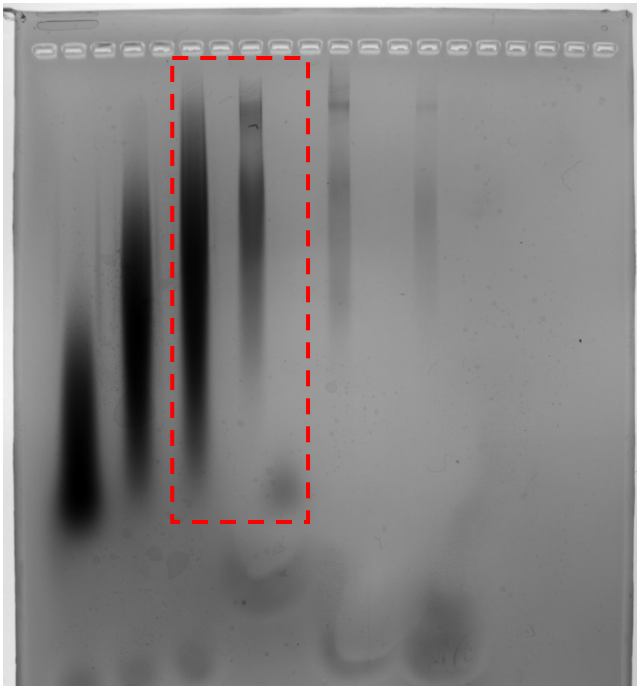

Supplementary Information Figure 1b

Extended Data Fig. 7b

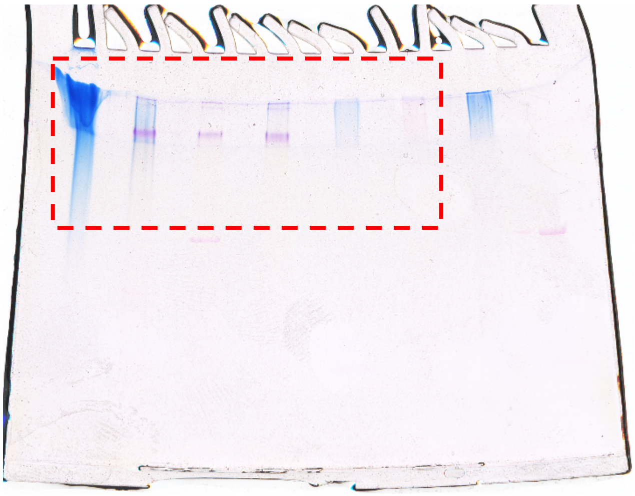

Extended Data Fig. 7g

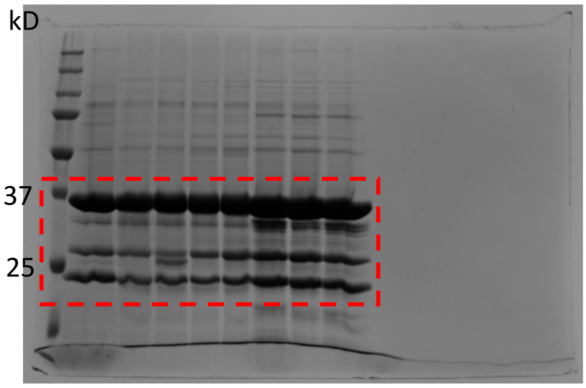

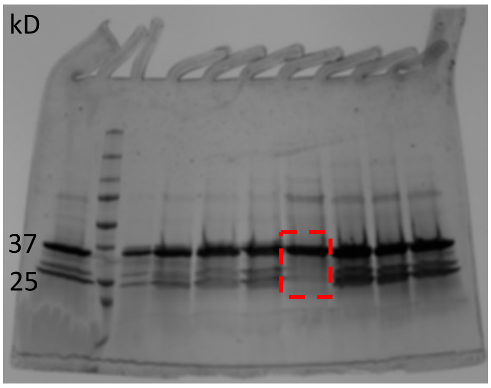

SI Figure 1 (a, b) | Uncropped gels presented in the specified ED Figures. Red dashed boxes indicate cutouts used in the figures.

Supplementary Information Figure 2

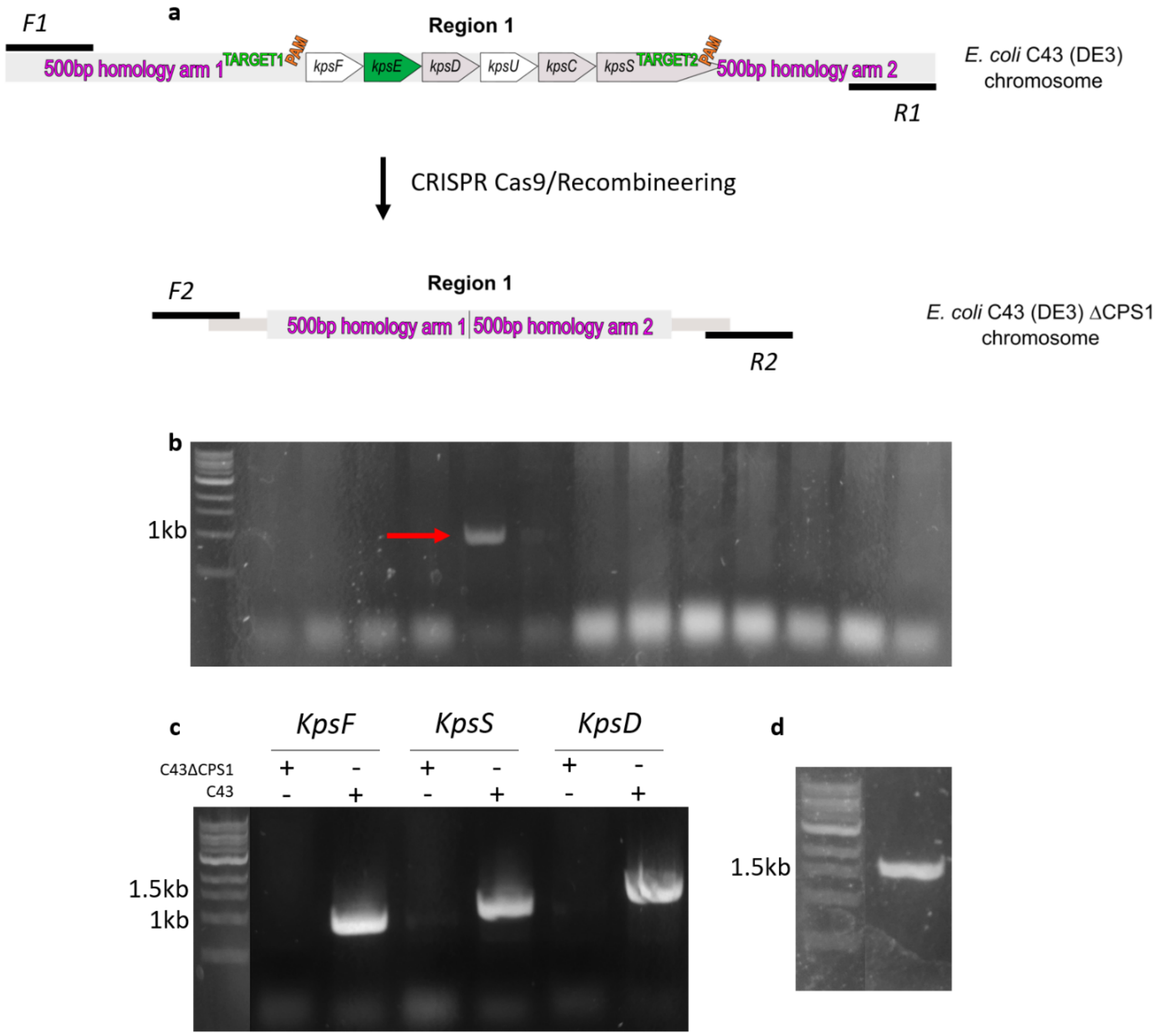

SI Figure 2 | CRISPR Cas9/Recombineering of the *E. coli* C43 genome. a, Deletion of Region 1 of the CPS gene cluster. **b**, Colony screening: genomic DNA amplified using primers directly flanking the homology arms (F1, R1) by colony PCR. The red arrow indicates a positive clone. **c**, Comparison of genes amplified by colony PCR from within the CPS1 gene cluster in C43 and C43ΔCPS1 cells. **d**, DNA fragment amplified from the genome of C43ΔCPS1 cells using primers annealing upstream and downstream of the deleted region (F2, R2). This fragment was purified and sequenced.

Supplementary Information Figure 3

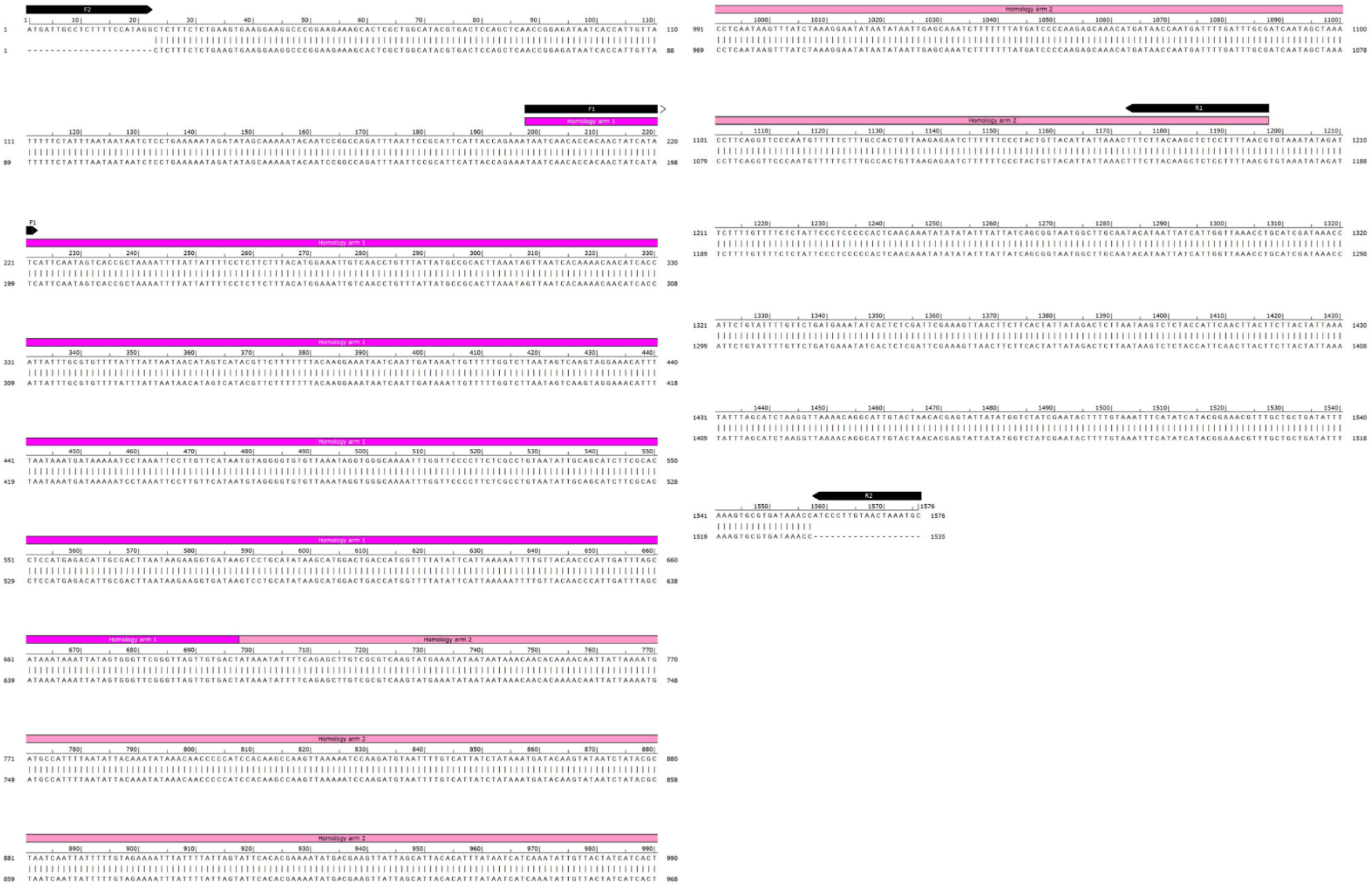

SI Figure 3 | Sequencing results of the fragment amplified from the C43ΔCPS1 genome. Homology arms are marked in magenta and pink; primers used in SI Fig. 2 are shown in black. Genomic sequence: top, sequencing results: bottom. Alignment generated in SnapGene, Dotmatics.

Supplementary Information Figure 4a

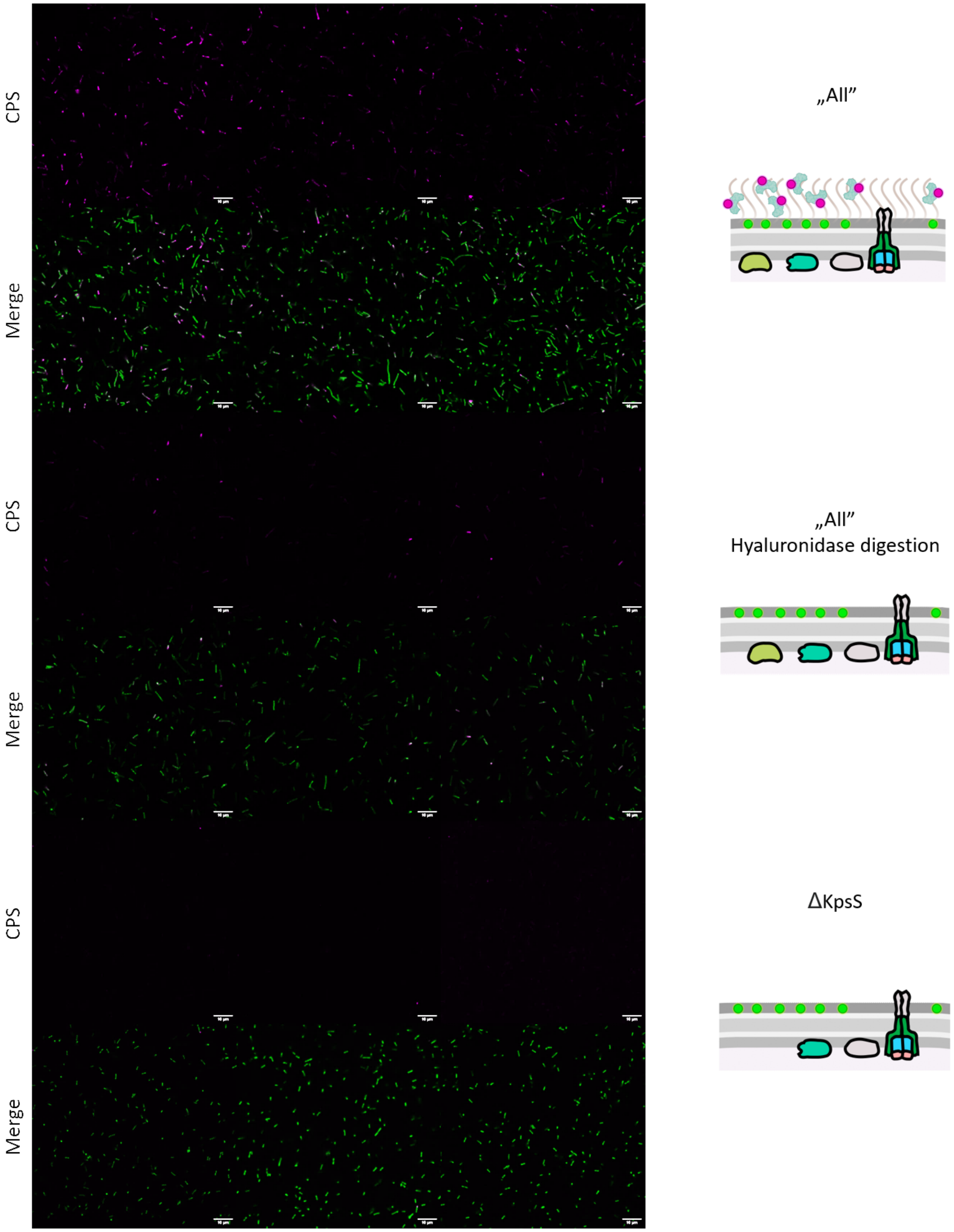

Supplementary Information Figure 4b

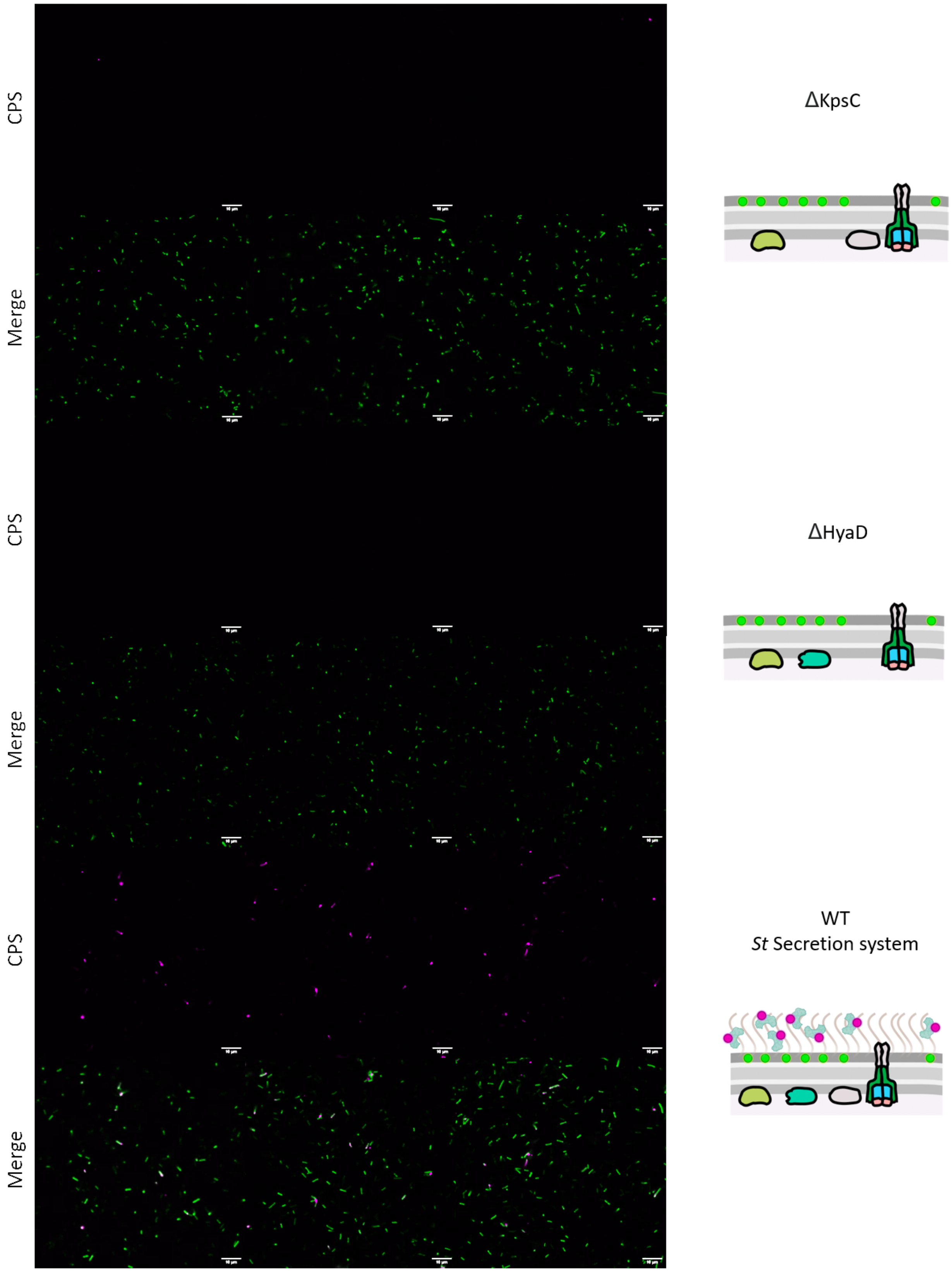

Supplementary Information Figure 4c

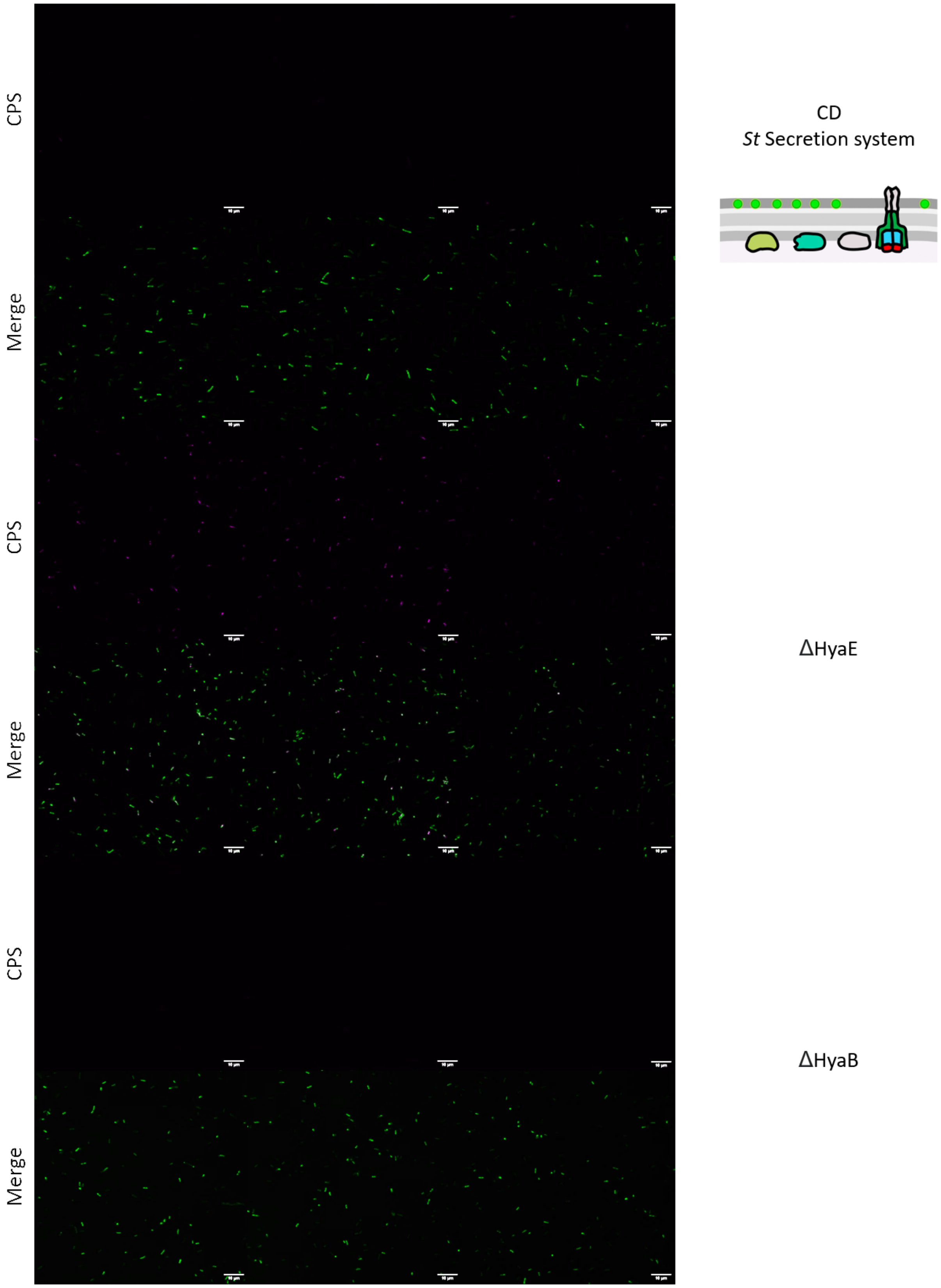

SI Figure 4 (a-c) | Three additional representative full fields of view of confocal images presented in Figure 1 and ED Figure 1. Parts of this figure were generated in BioRender.com. Scalebar: 10µm.

Supplementary Information Figure 5

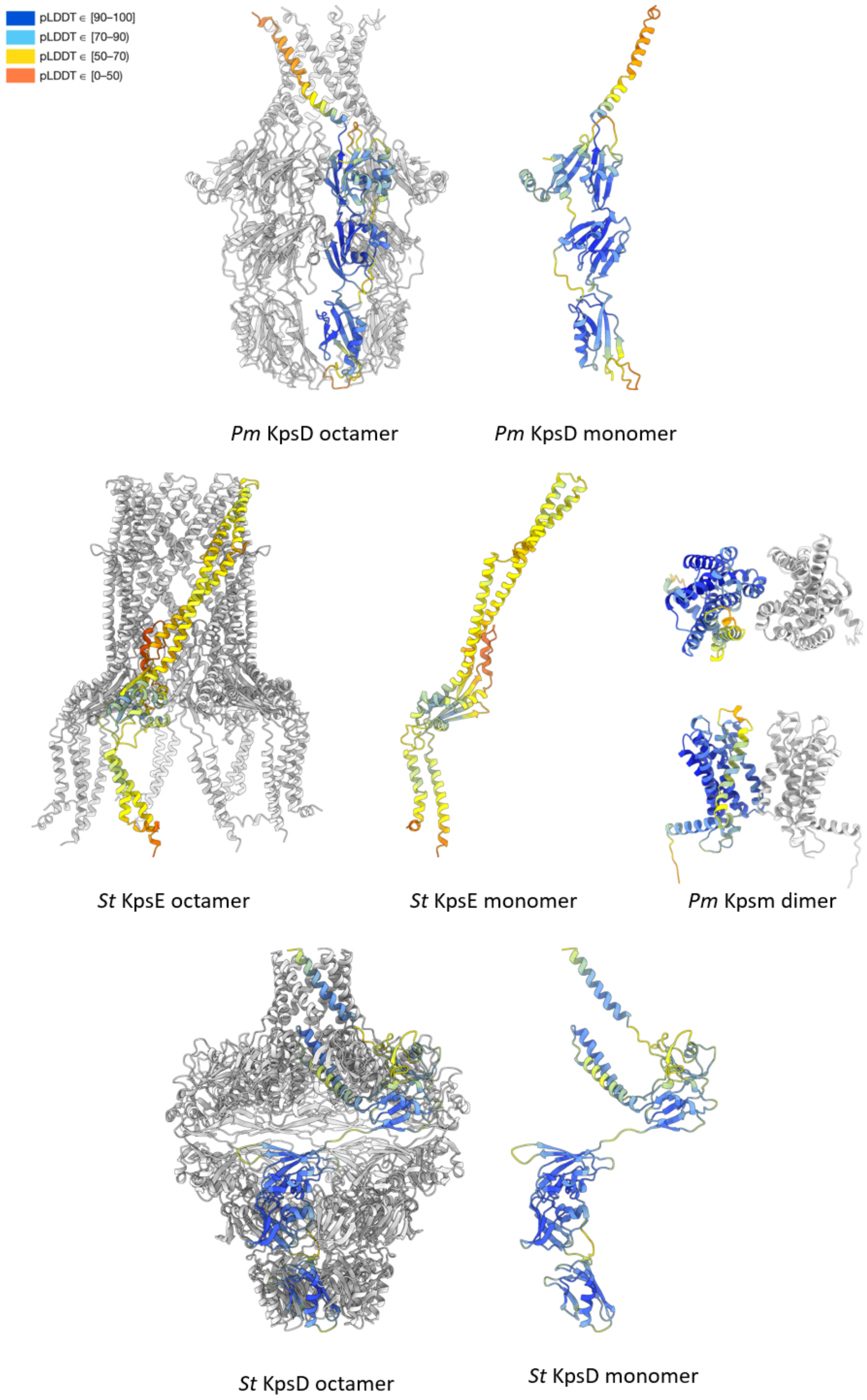

SI Figure 5 | pLDDT (predicted local distance difference test) values for all AlphaFold2 predicted models.

Supplementary Information Figure 6

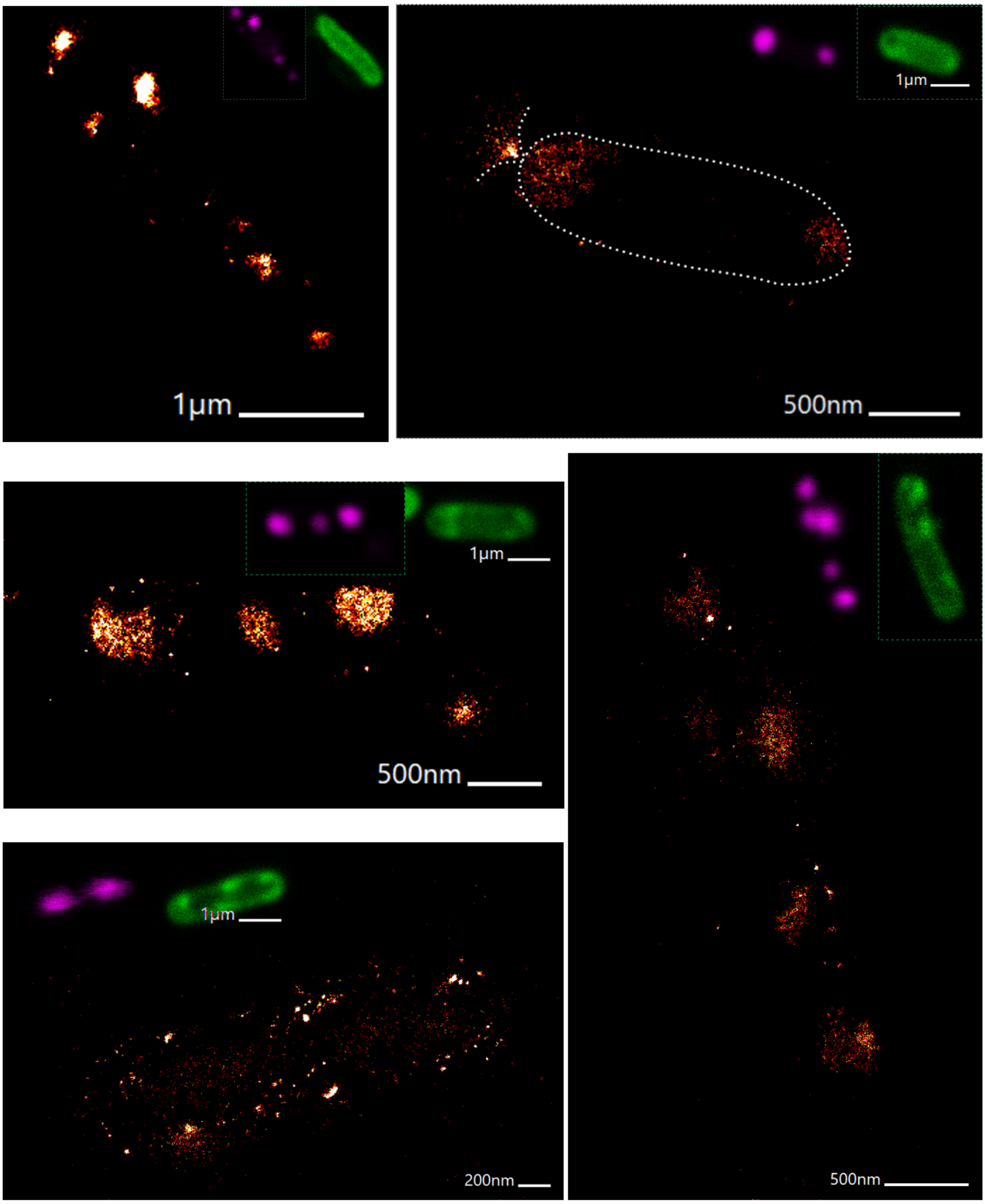

SI Figure 6 | Diversity of CPS puncta morphologies observed using Minflux nanoscopy. Five different cells are shown. On the confocal level, the CPS formations resemble distinct puncta. Top right: Volcano-like CPS scattering and the cell outline indicated with a dashed line.

Supplementary Information Figure 7

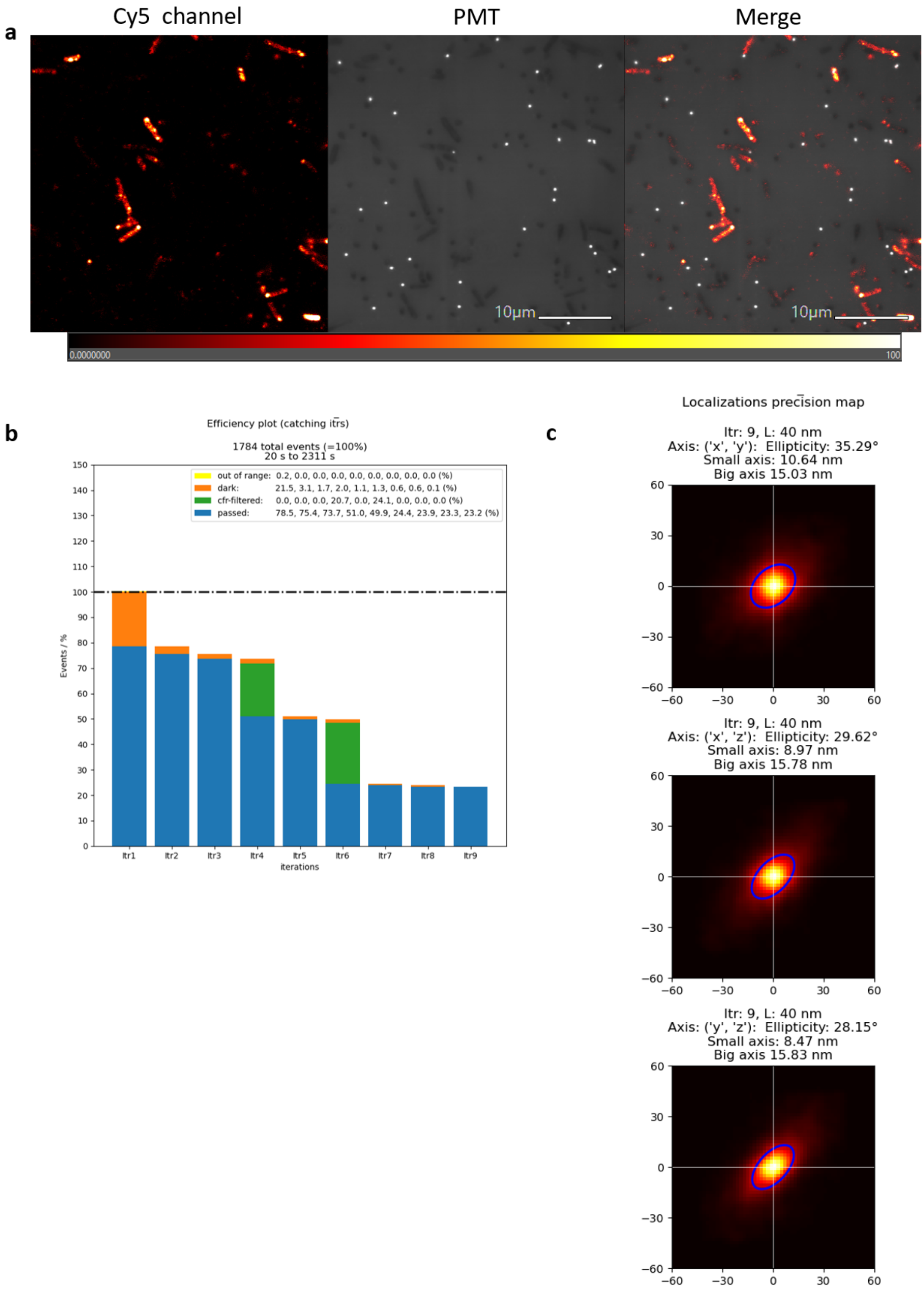

SI Figure 7 | 3D Minflux labeling of an encapsulated cell with the volcano-like CPS formation. a, Confocal fields of view for the CPS imaging. The signal intensity detected in the Cy5 channel is scaled to 100. **b** and **c**, Minflux dataset collection parameters for the 3D experiment. Efficiency plot of caught fluorophores (b) and raw burst precision estimates (c).

Supplementary Information Figure 8

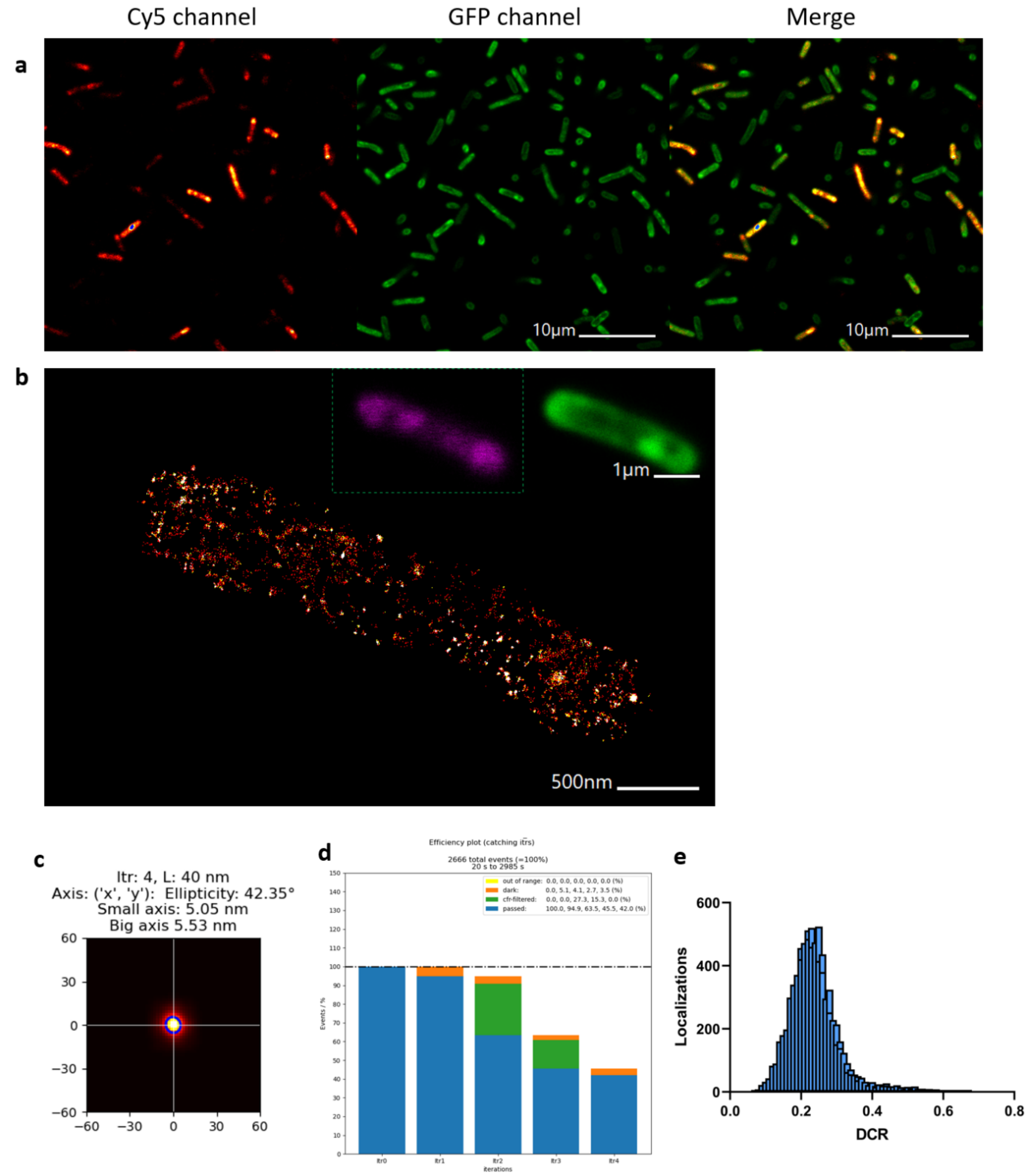

SI Figure 8 | Minflux nanoscopy of a cell expressing „All" CPS biosynthetic components and labeled with ^{Flux680}CBM70. a, Confocal fields of view for this single fluorophore experiment. The intensity of the signal detected in the Cy5 channel is scaled to 100. **b**, Representative Minflux nanoscopy of CPS of an encapsulated cell using CBM70:Flux680, inlet: zoomed in confocal image of the cell used for Minflux. **c** and **d**, Representative Minflux dataset collection parameters. Efficiency plot of caught fluorophores (c) and raw burst precision estimates (d). **e**, DCR histogram.

Supplementary Information Figure 9

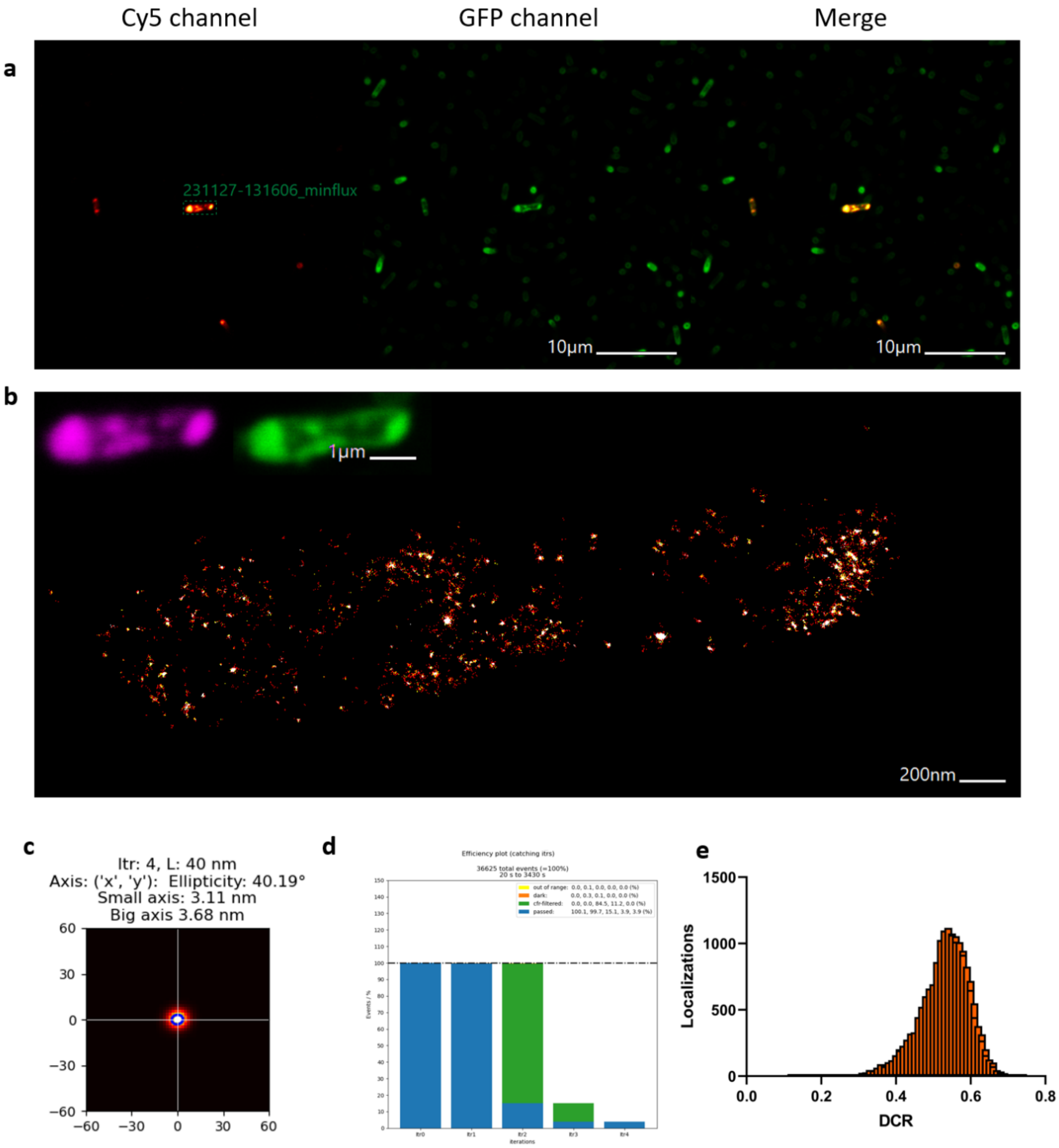

SI Figure 9 | Minflux nanoscopy of LPS in cells expressing „All" CPS biosynthetic components metabolically labelled with KDO-azide and labeled with AZ647 DBCO. a, Confocal fields of view for this single fluorophore experiment. The intensity of the signal detected in the Cy5 channel is scaled to 100. **b**, Representative Minflux nanoscopy of LPS of an encapsulated cell using KDO-azide/DBCO-AF647, inlet: zoomed in confocal image of the cell used for Minflux. **c** and **d**, Representative Minflux dataset collection parameters. Efficiency plot of caught fluorophores (c) and raw burst precision estimates (d). **e**, DCR histogram.

Supplementary Information Figure 10

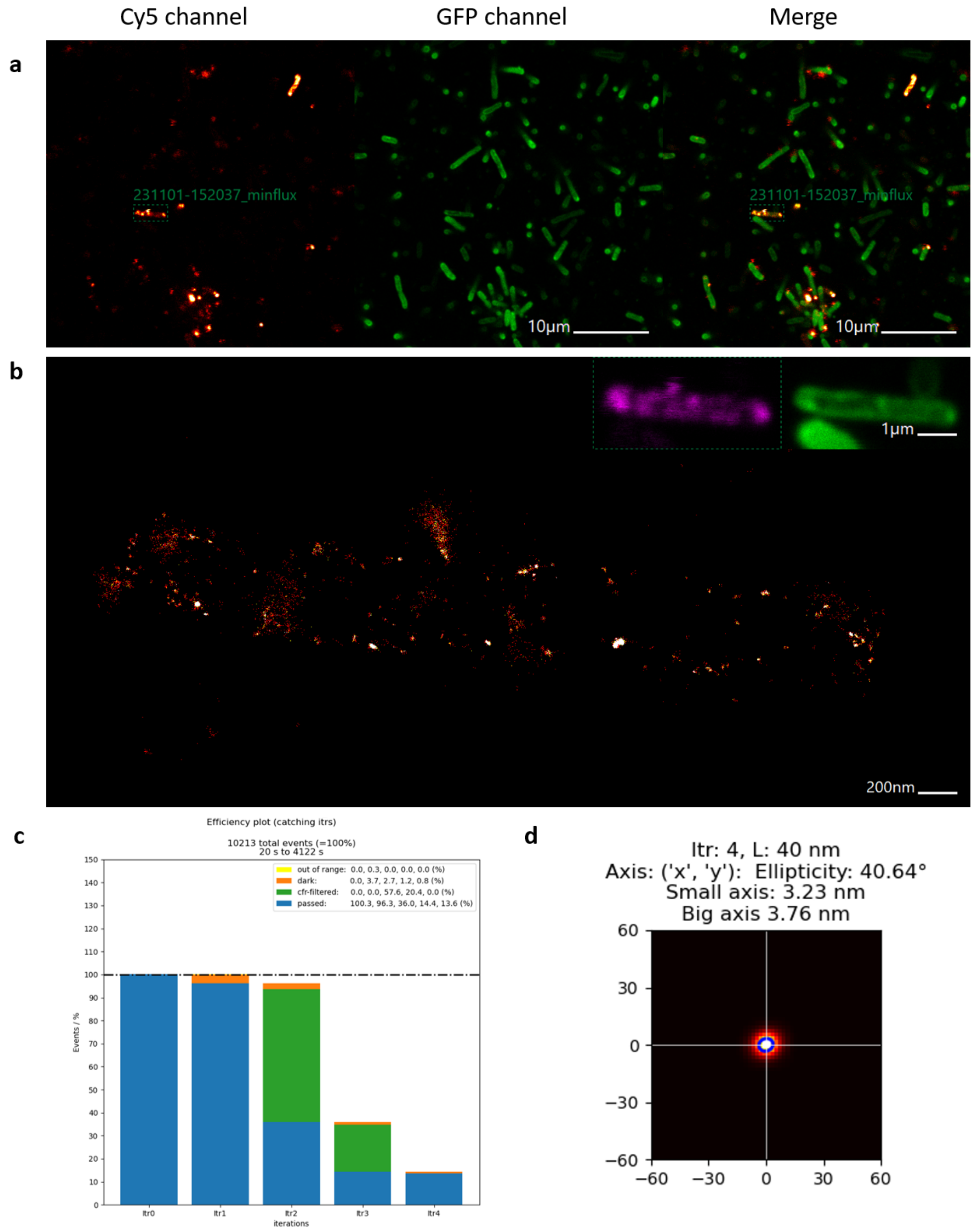

SI Figure 10 | Minflux labeling of CPS and LPS. a, Confocal fields of view for the dual fluorophore experiment presented in Extended Data Fig. 1k and l. The signal intensity in the Cy5 channel is scaled to 100. **b**, Minflux localizations of the combination of fluorophores, inlet: zoomed in confocal image of the cell used for Minflux. **c** and **d,** Minflux dataset collection parameters for experiment presented in Extended Data Fig. 1k and l. Efficiency plot of caught fluorophores (c) and raw burst precision estimates (d).

Supplementary Information Figure 11

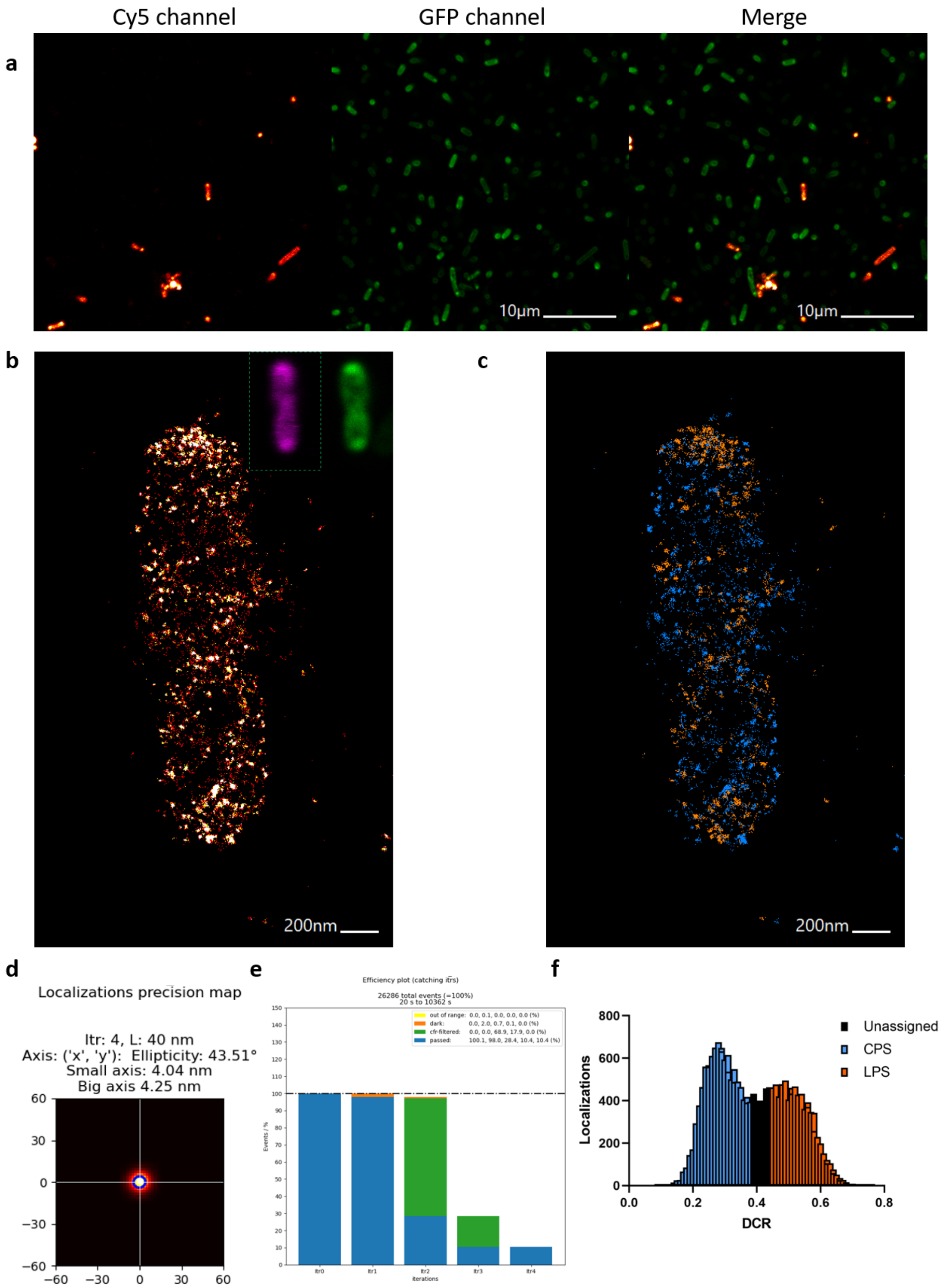

SI Figure 11 | Second example of a two-color Minflux dataset of a cell expressing „All" CPS biosynthetic components and labeled as in ED Figure 1k. a, Confocal field of view for this experiment. **b**, Minflux localizations of the combination of fluorophores, inlet: zoomed in confocal image of the cell used for Minflux. Volcano-like CPS scattering and the cell outline indicated with a dashed line. **c** and **d**, Minflux localizations colored according to the DCR values. Localizations were rendered with the pixel size based on the localization precision map shown in panel. **e** and **f**, Efficiency plot of caught fluorophores (e) and DCR histogram (f).

Supplementary Information Figure 12

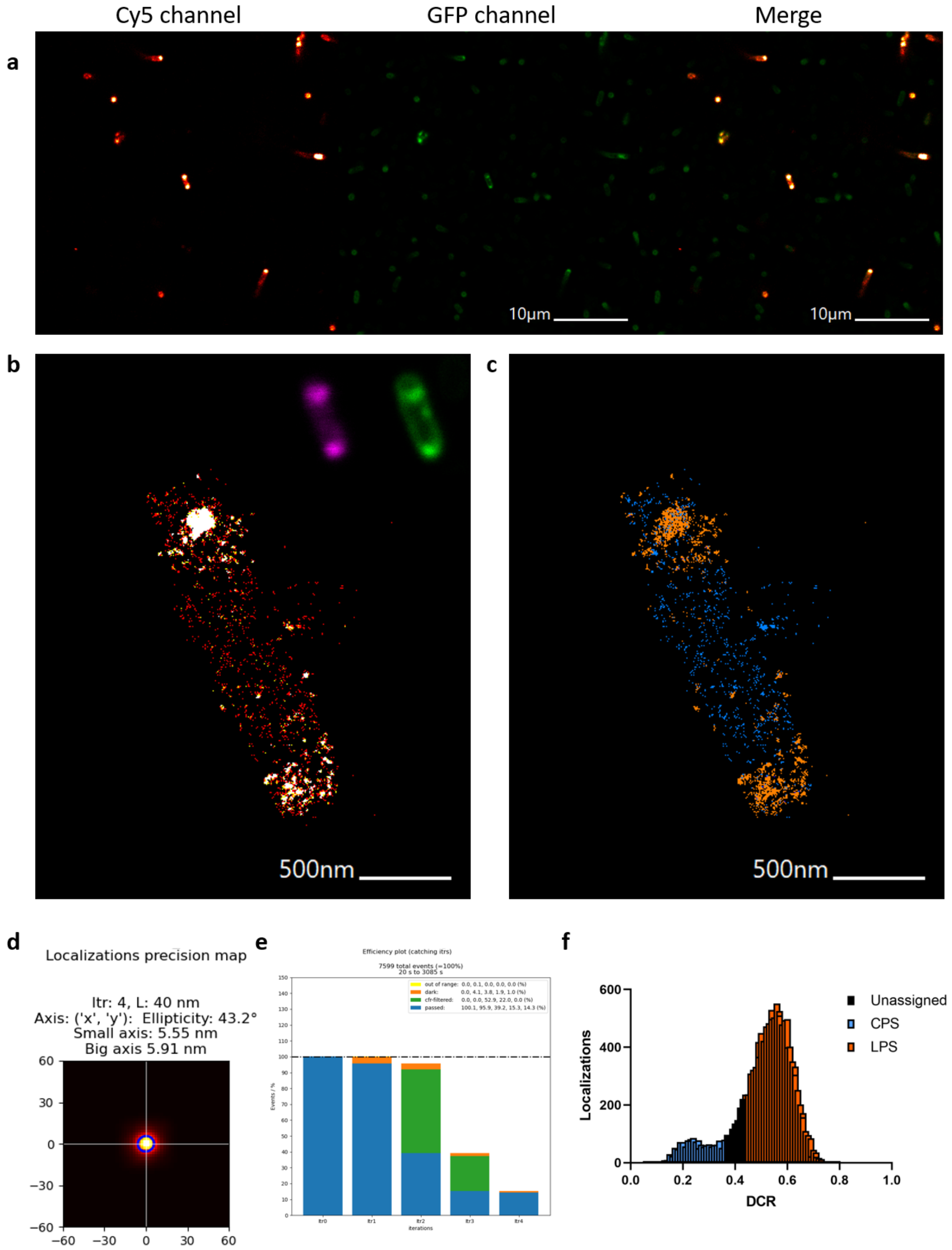

SI Figure 12 | Third example of a two-color Minflux dataset of a cell expressing „All" CPS biosynthetic components and labeled as in ED Figure 1k. a, Confocal field of view for this experiment. **b**, Minflux localizations of the combination of fluorophores, inlet: zoomed in confocal image of the cell used for Minflux. Volcano-like CPS scattering and the cell outline indicated with a dashed line. **c** and **d**, Minflux localizations colored according to the DCR values. Localizations were rendered with the pixel size based on the localization precision map shown in panel. **e** and **f**, Efficiency plot of caught fluorophores (e) and DCR histogram (f).

Supplementary Information Figure 13

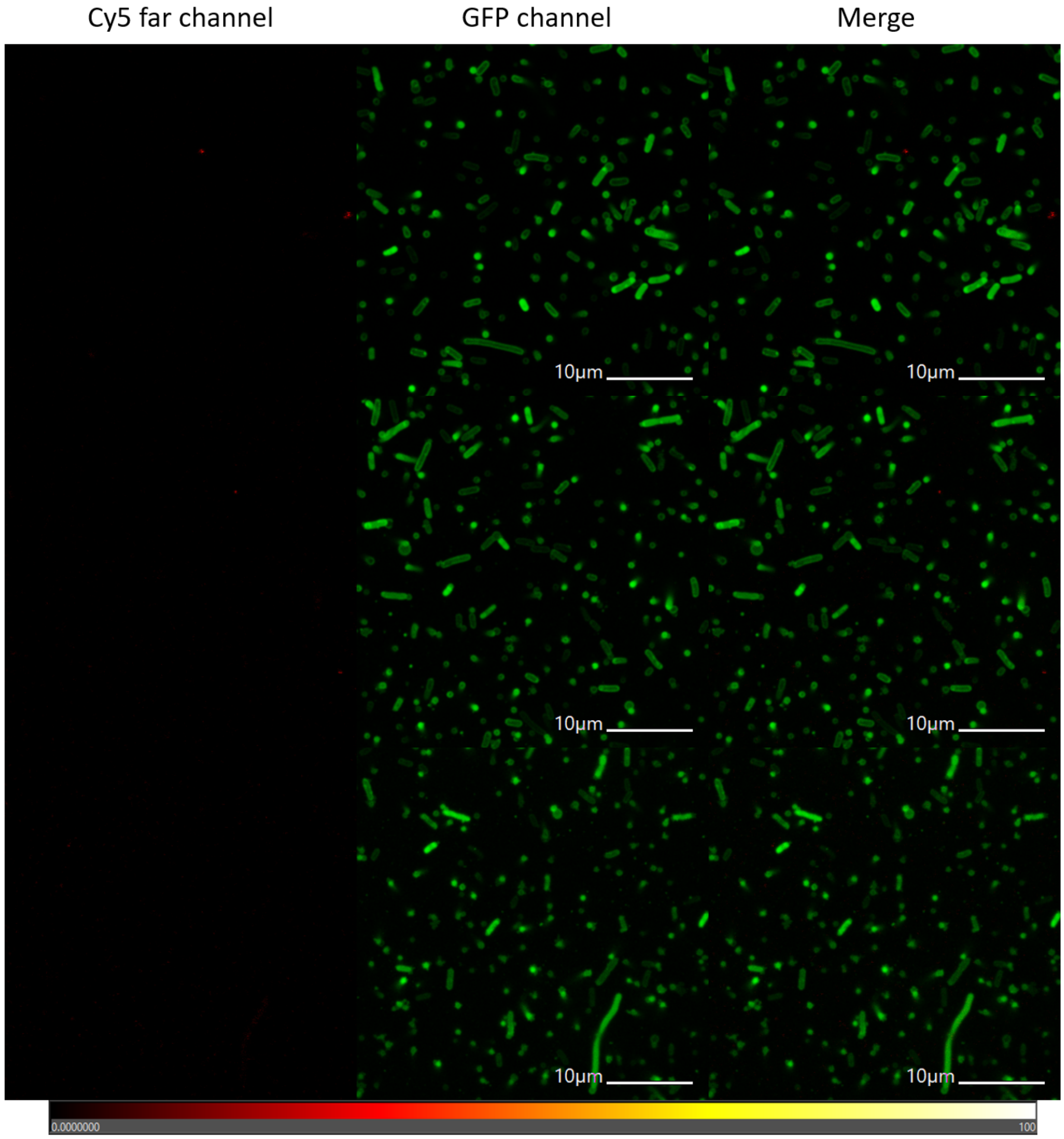

SI Figure 13 | ^{Alexa680}Streptavidin does not bind to cells lacking Strep-tagged *Pm* KpsD. Cells expressing *St* KpsMT-KpsE-KpsD were treated as for cells shown in Figure 1 and Extended Data Fig. 1. Little to no signal in the Cy5 far channel was detected in three consecutive trials. The signal intensity detected in the Cy5 channel is scaled to 100 for all the examples shown.

Supplementary Information Figure 14

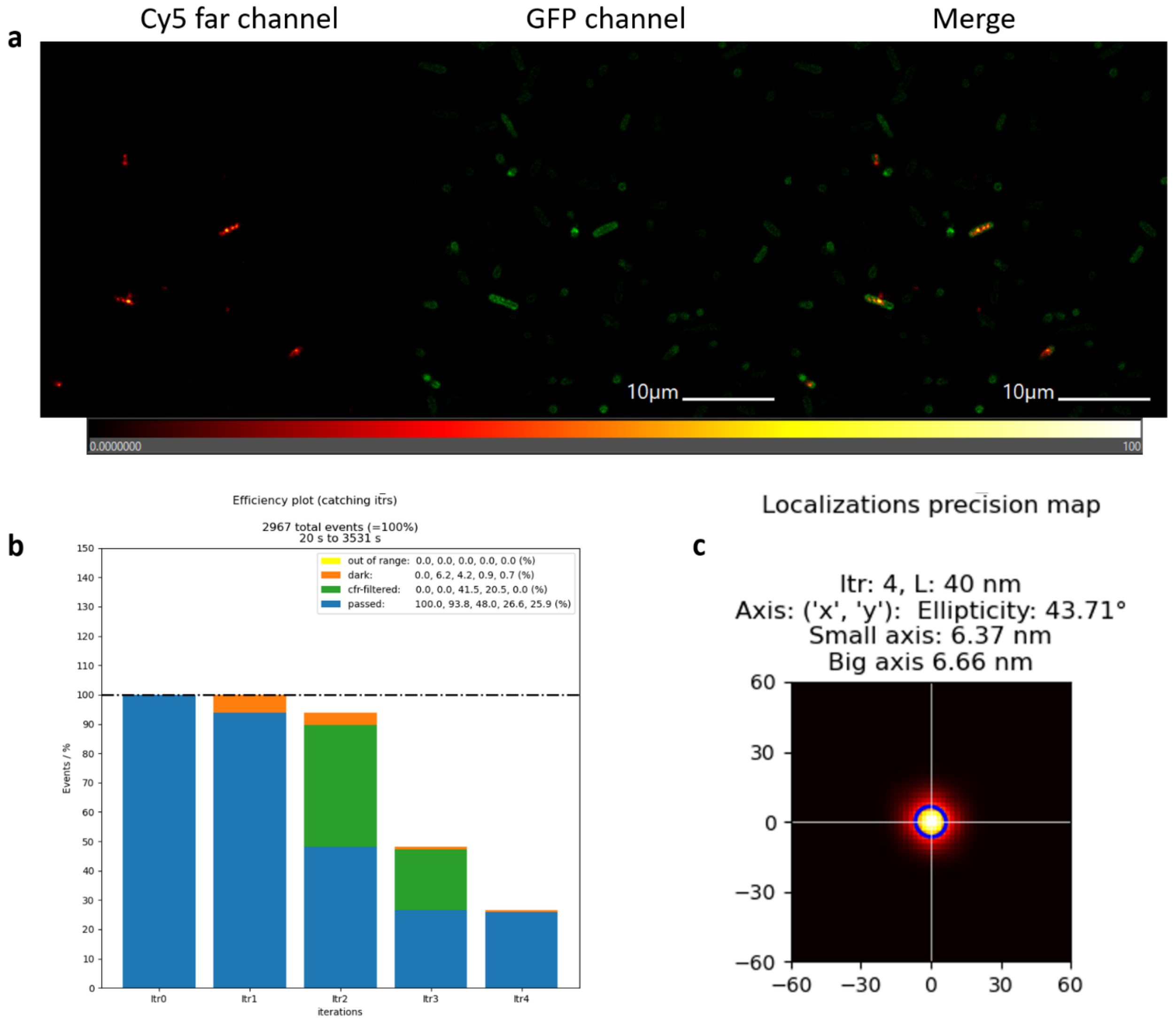

SI Figure 14 | Minflux labeling of *Pm* KpsD. a, Confocal fields of view for the single fluorophore experiment presented in Extended Data Fig. 1g. *Pm* KpsD was detected with ^{Alexa680}Streptavidin. The signal intensity detected in the Cy5 channel is scaled to 100. **b** and **c**, Minflux dataset collection parameters for experiment presented in Extended Data Fig. 1g. Efficiency plot of caught fluorophores (b) and raw burst precision estimates (c).

Supplementary Information Figure 15

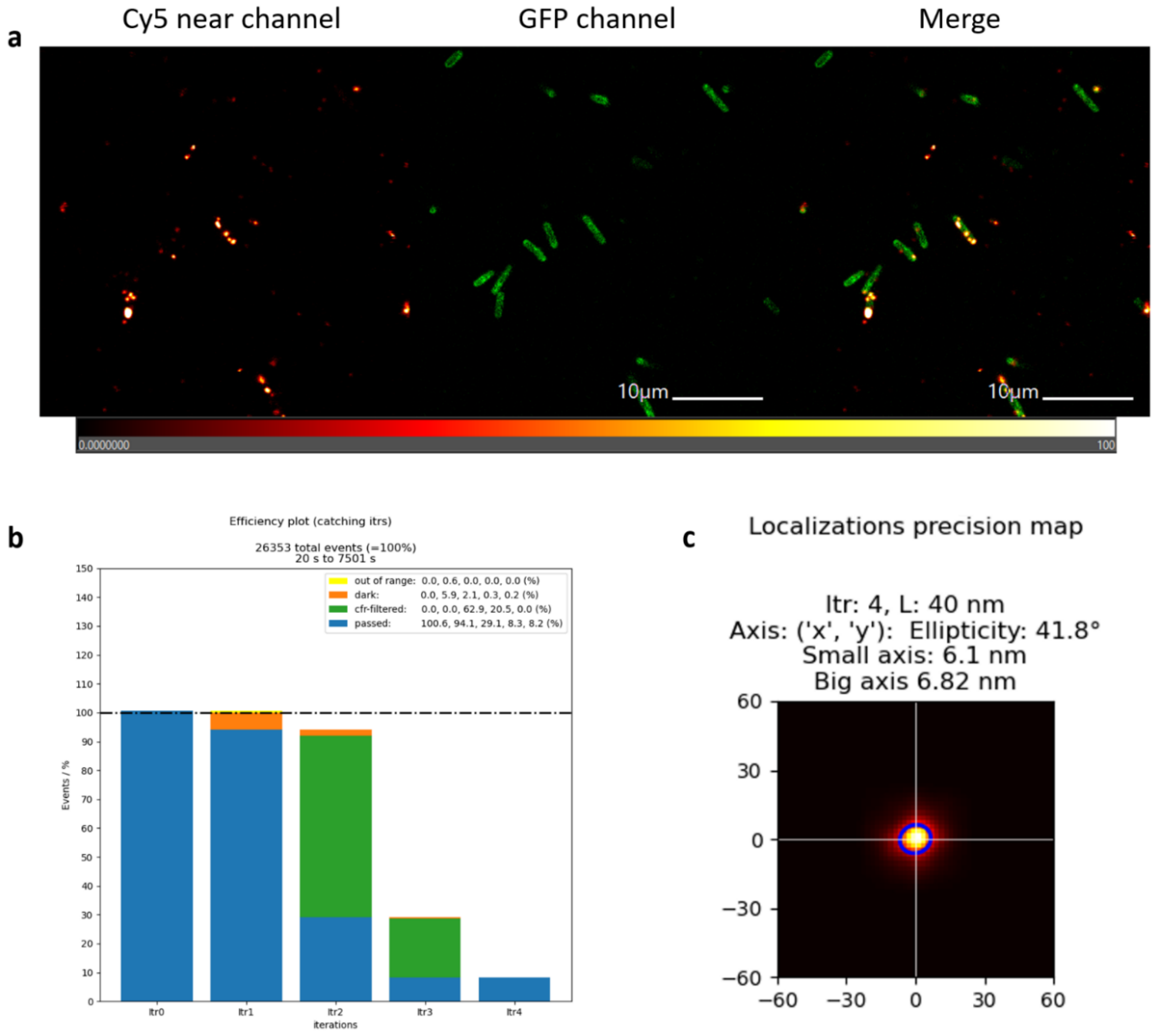

SI Figure 15 | Minflux labeling of CPS. a, Confocal fields of view for single fluorophore experiment presented in Extended Data Fig. 1h. The signal intensity in the Cy5 channel is scaled to 100. **b** and **c**, Minflux dataset collection parameters for experiment presented in Extended Data Fig. 1h. Efficiency plot of caught fluorophores (b) and raw burst precision estimates (c).

Supplementary Information Figure 16

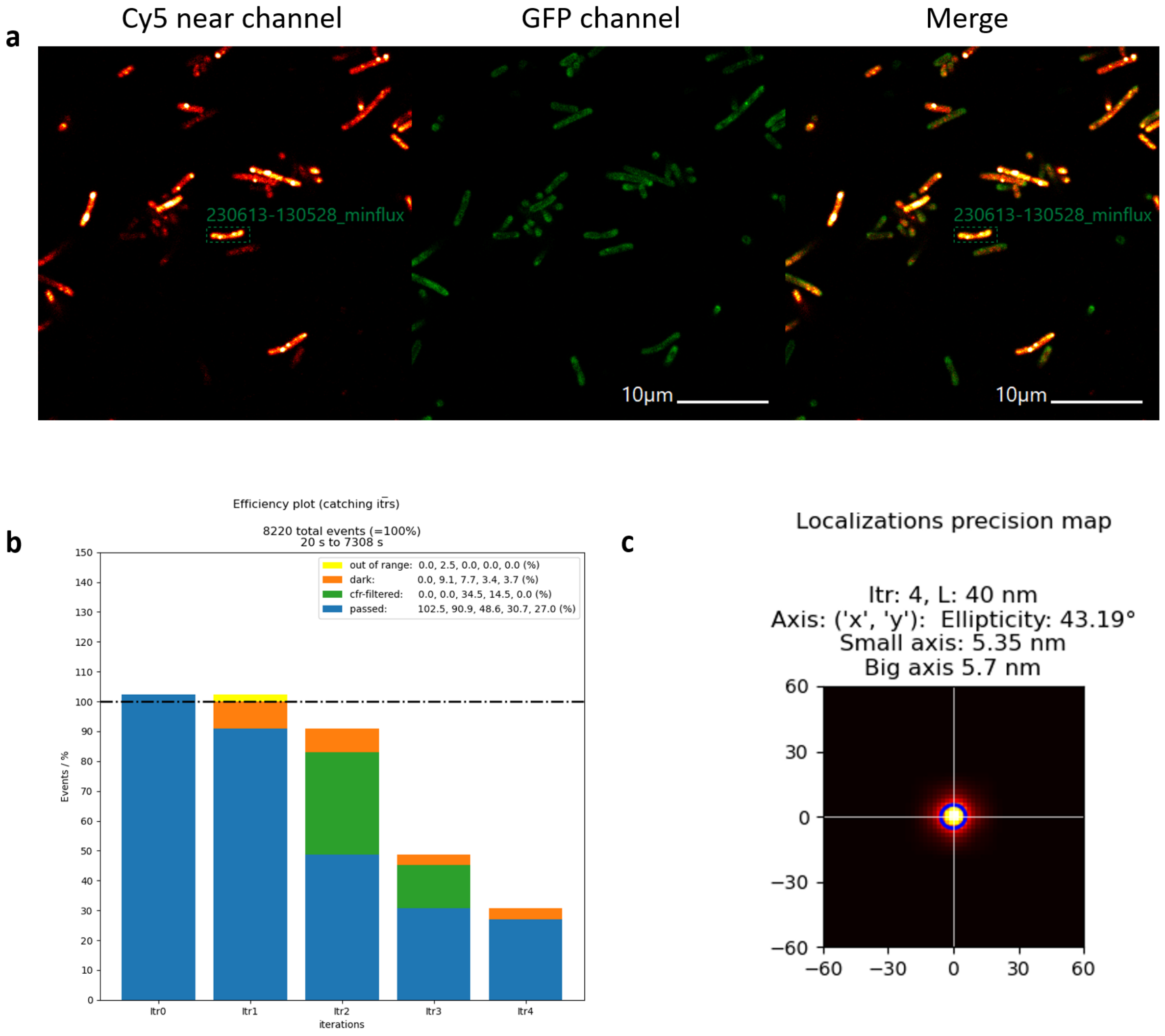

SI Figure 16 | Minflux labeling of CPS and *Pm* KpsD. a, Confocal fields of view for the dual fluorophore experiment presented in Fig. 1f and Extended Data Fig. 1i and j. The signal intensity in the Cy5 channel is scaled to 100. **b** and **c,** Minflux dataset collection parameters for experiment presented in Fig. 1f and Extended Data Fig. 1i and j. Efficiency plot of caught fluorophores (b) and raw burst precision estimates (c).

Supplementary Information Figure 17

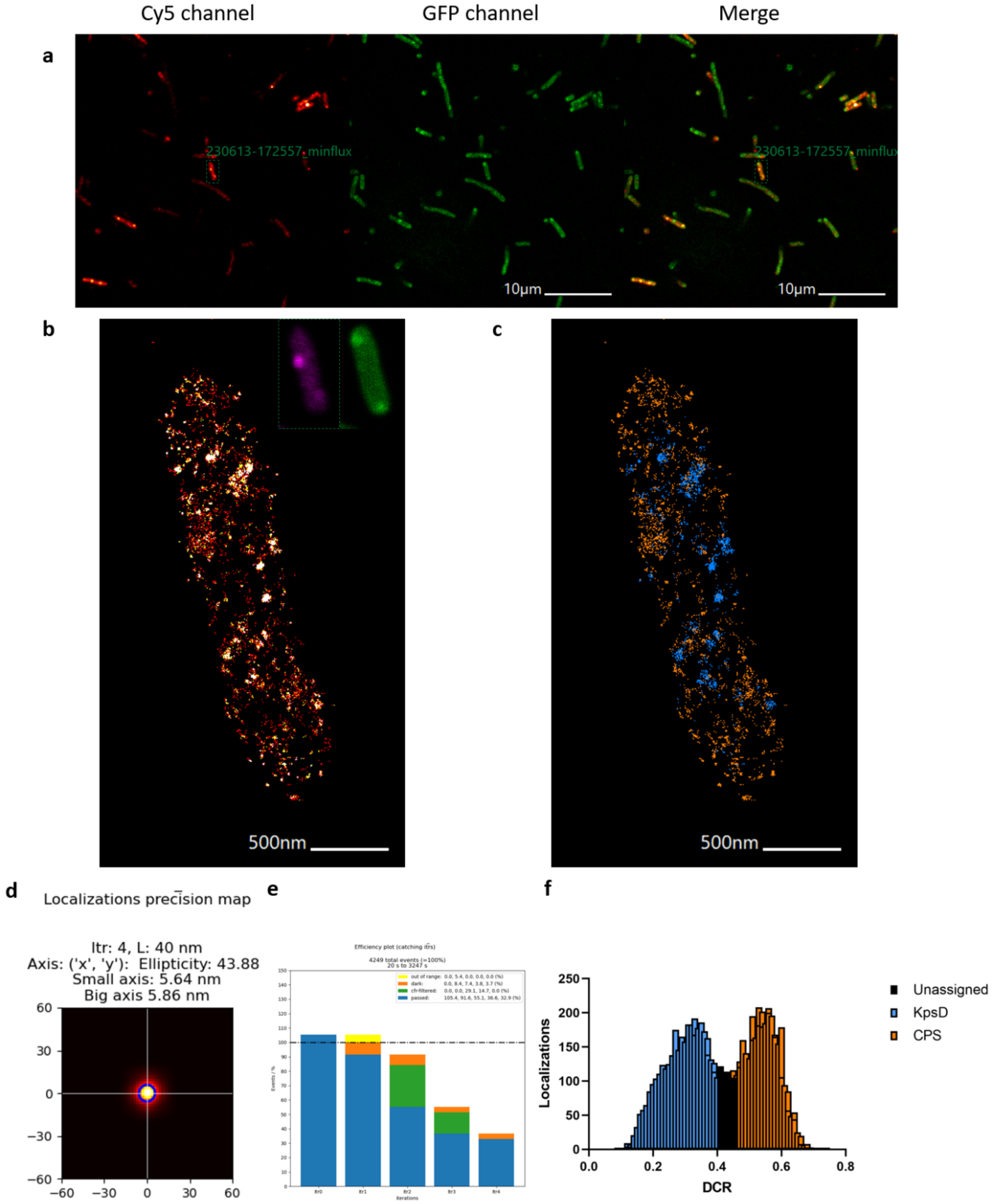

SI Figure 17 | Second example of a two-color Minflux dataset of a cell expressing „All" CPS biosynthetic components and labeled as in Figure 1f. a, Confocal field of view for this experiment. **b**, Minflux localizations of the combination of fluorophores, inlet: zoomed in confocal image of the cell used for Minflux. **c** and **d**, Minflux localizations colored according to the DCR values. Localizations were rendered with the pixel size based on the localization precision map shown in panel d. **e** and **f**, Efficiency plot of caught fluorophores (e) and DCR histogram (f).

Supplementary Information Figure 18

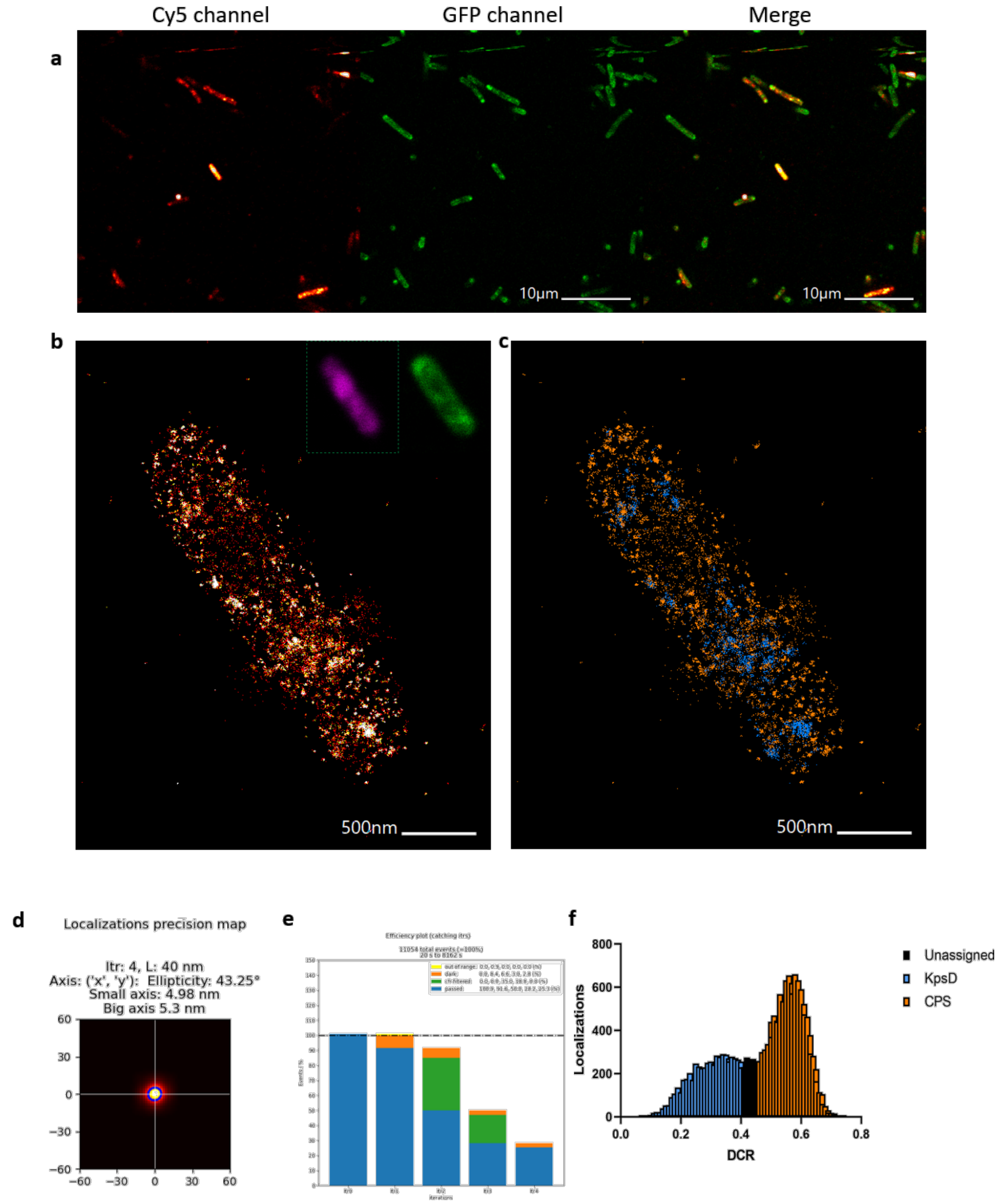

SI Figure 18 | Third example of a two-color Minflux dataset of a cell expressing „All" CPS biosynthetic components and labeled as in Figure 1f. a, Confocal field of view for this experiment. **b**, Minflux localizations of the combination of fluorophores, inlet: zoomed in confocal image of the cell used for Minflux. **c** and **d**, Minflux localizations colored according to the DCR values. Localizations were rendered with the pixel size based on the localization precision map shown in panel. **e** and **f**, Efficiency plot of caught fluorophores (e) and DCR histogram (f).

Supplementary Information Figure 19

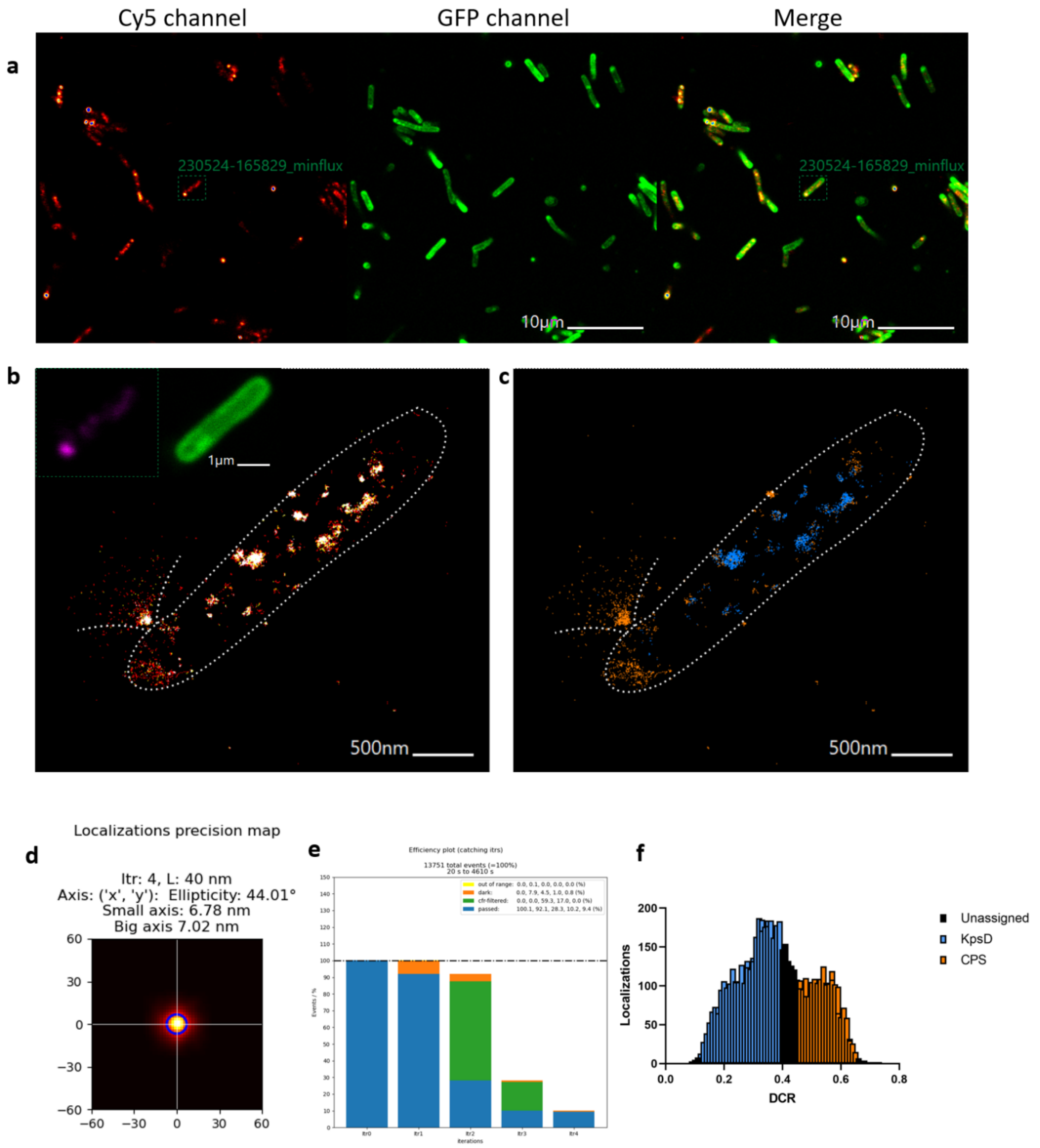

SI Figure 19 | Fourth example of a two-color Minflux dataset of a cell expressing „All" CPS biosynthetic components and labeled as in Figure 1f. a, Confocal field of view for this experiment. **b**, Minflux localizations of the combination of fluorophores, inlet: zoomed in confocal image of the cell used for Minflux. Volcano-like CPS scattering and the cell outline indicated with a dashed line. **c** and **d**, Minflux localizations colored according to the DCR values. Localizations were rendered with the pixel size based on the localization precision map shown in panel. **e** and **f**, Efficiency plot of caught fluorophores (e) and DCR histogram (f).

Supplementary Information Figure 20

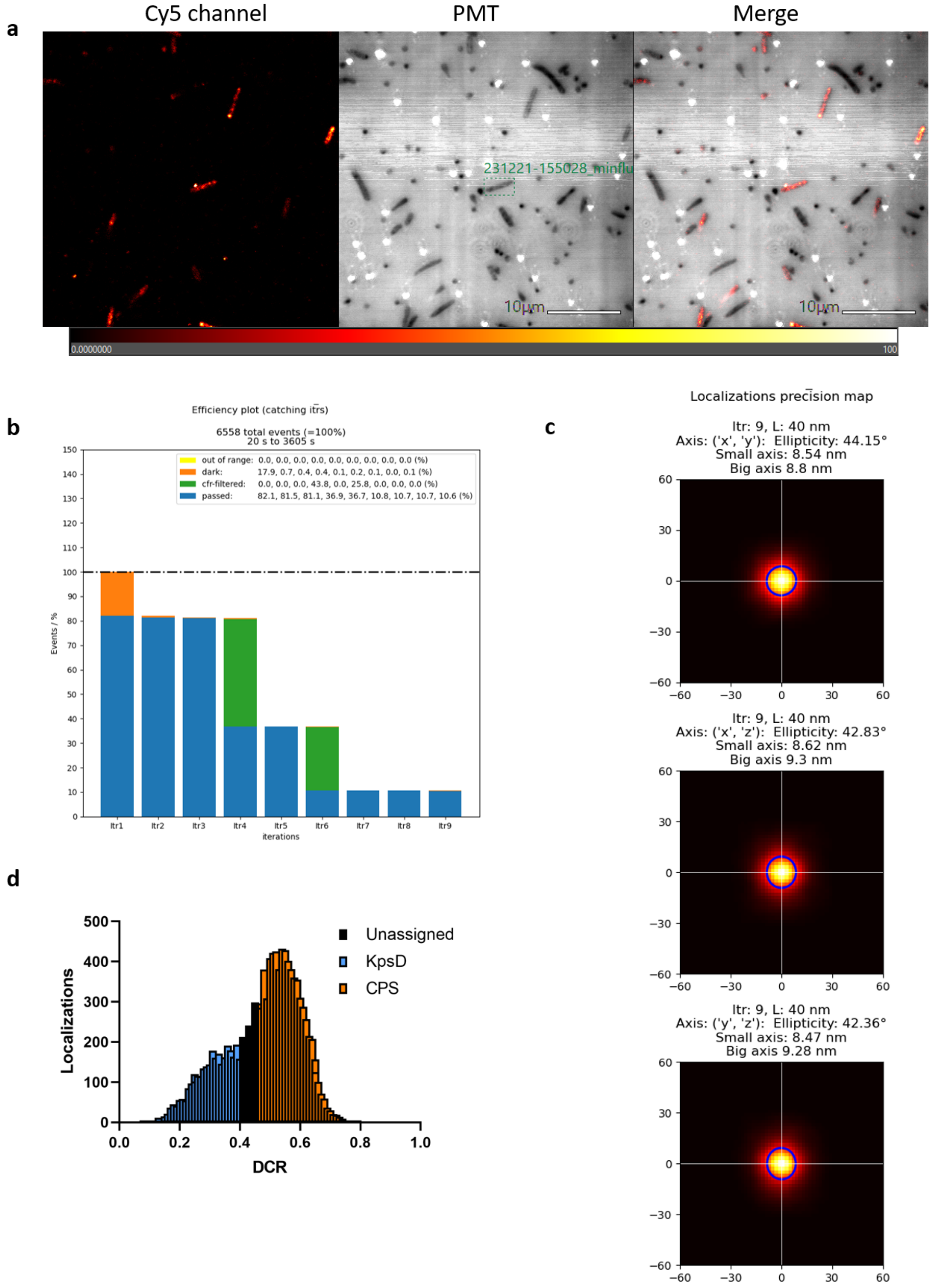

SI Figure 20 | 2 color 3D Minflux nanoscopy of a cell expressing „All" CPS biosynthetic components and labeled as in Figure 1f. a, Confocal fields of view for this experiment. The signal intensity detected in the Cy5 channel is scaled to 100. **b** and **c**, Minflux dataset collection parameters for the 3D experiment. Efficiency plot of caught fluorophores (b) and raw burst precision estimates (c). **d,** DCR histogram.

Supplementary Information Figure 21

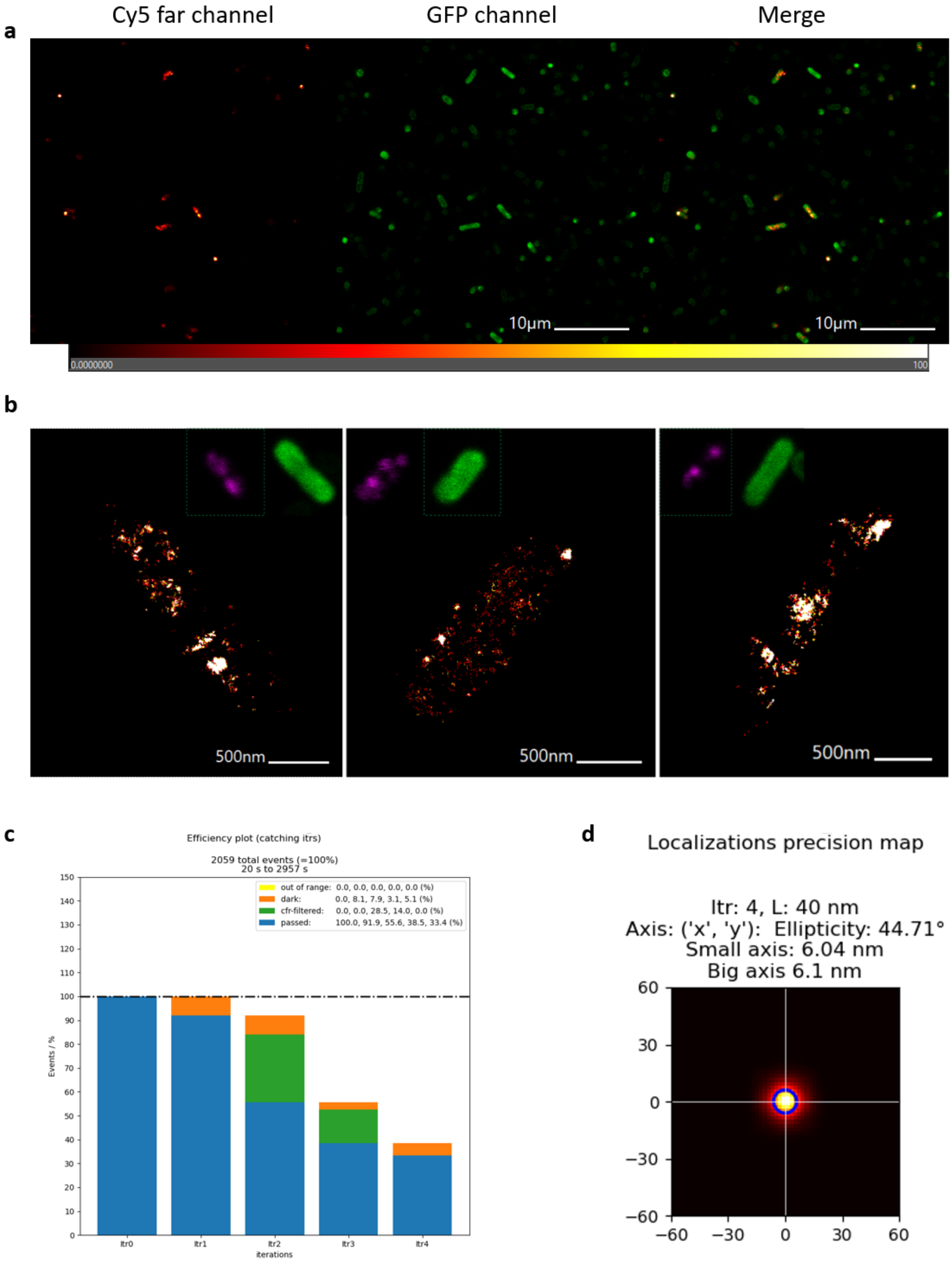

SI Figure 21 | Minflux nanoscopy of ΔHyaD cells labeled with ^{Alexa680}Streptavidin. a, Confocal fields of view for single fluorophore experiments done on ΔHyaD cells. The intensity of the signal detected in the Cy5 channel is scaled to 100. **b**, Three representative Minflux datasets of ΔHyaD cells labeled with ^{Alexa680}Streptavidin. **c** and **d**, Representative Minflux dataset collection parameters for the ΔHyaD datasets. Efficiency plot of caught fluorophores (c) and raw burst precision estimates (d).

Supplementary Information Figure 22

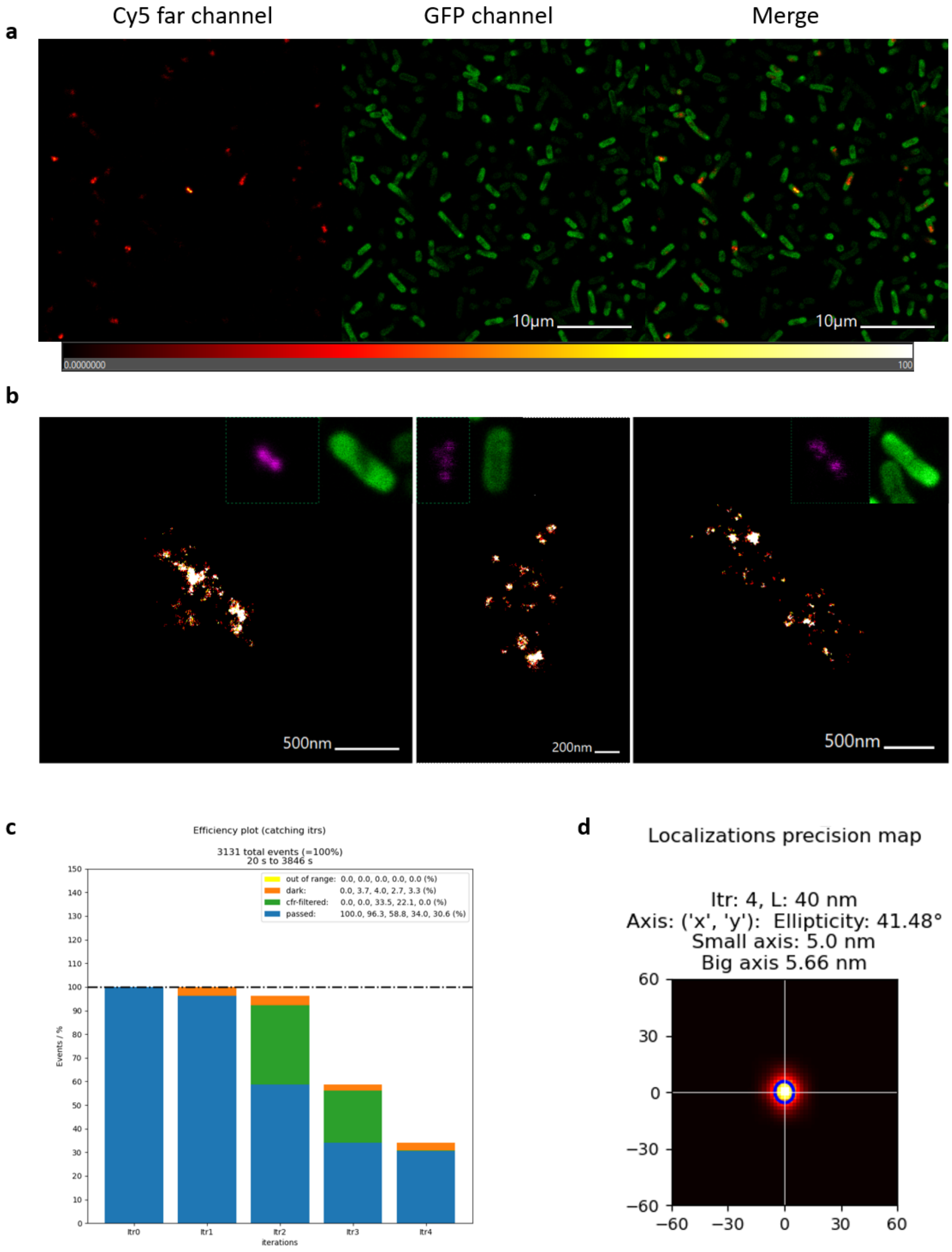

SI Figure 22 | Minflux nanoscopy of ΔKpsE cells labeled with ^{Alexa680}Streptavidin. a, Confocal fields of view for single fluorophore experiments done on ΔKpsE cells. The intensity of the signal detected on Cy5 channel is scaled to 100. **b**, Three representative examples of Minflux datasets of ΔKpsE cells labeled with ^{Alexa680}Streptavidin. **c** and **d**, Representative Minflux dataset collection parameters for ΔKpsE datasets. Efficiency plot of caught fluorophores (c) and raw burst precision estimates (d).

Supplementary Information Figure 23a

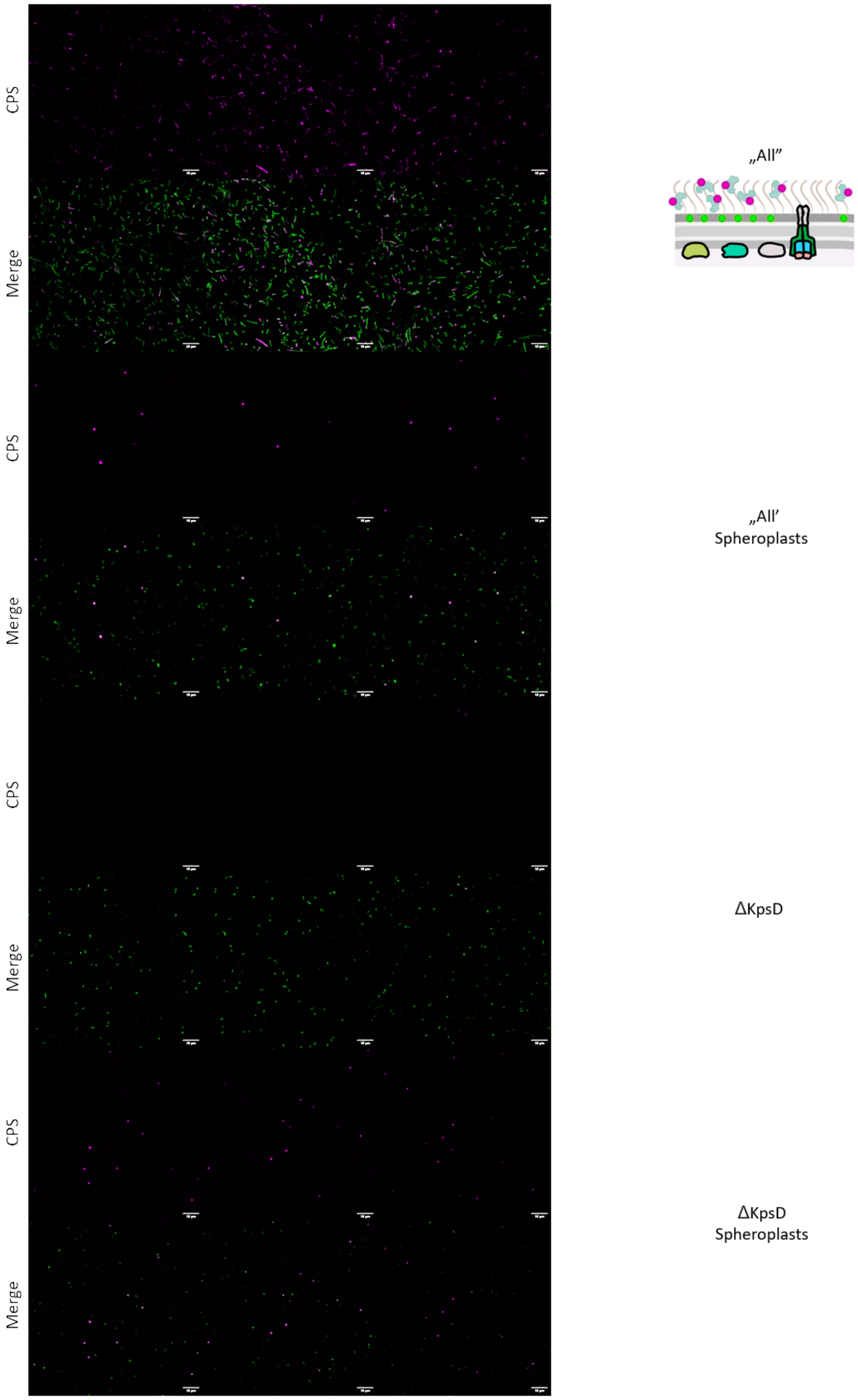

Supplementary Information Figure 23b

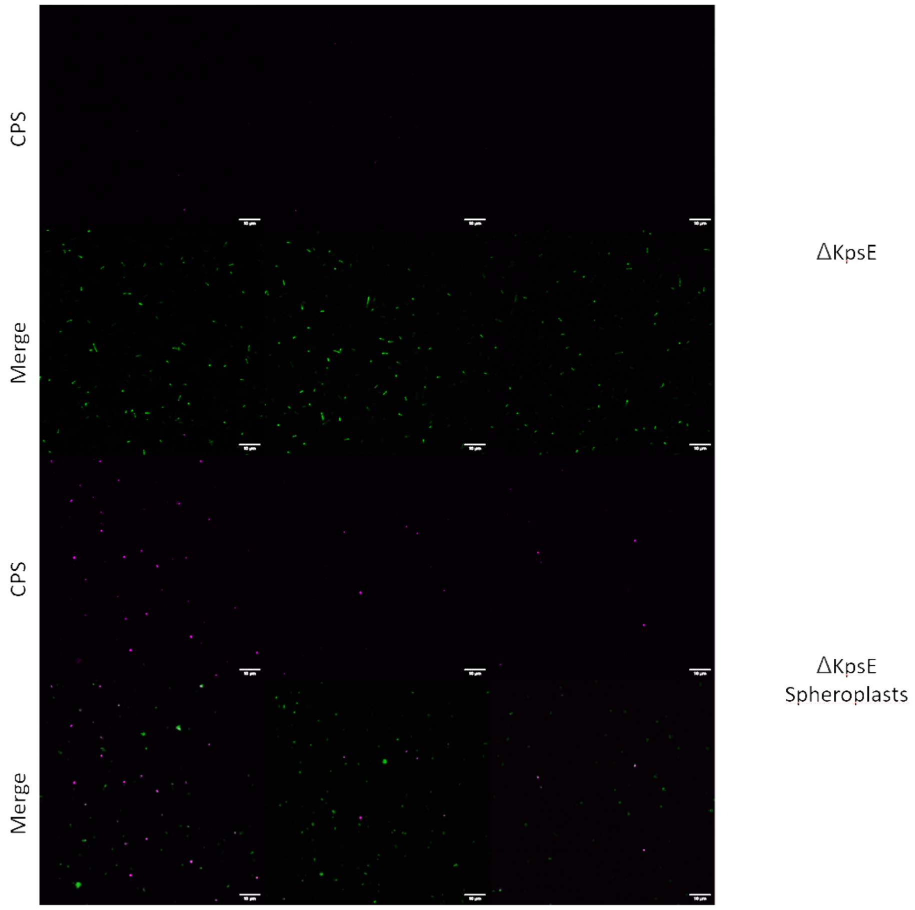

SI Figure 23 (a, b) | Representative full fields of view and additional examples of confocal imaging presented in Figure 1g. Parts of this figure were generated in BioRender.com. Scalebar: 10µm.

Supplementary Information Figure 24a

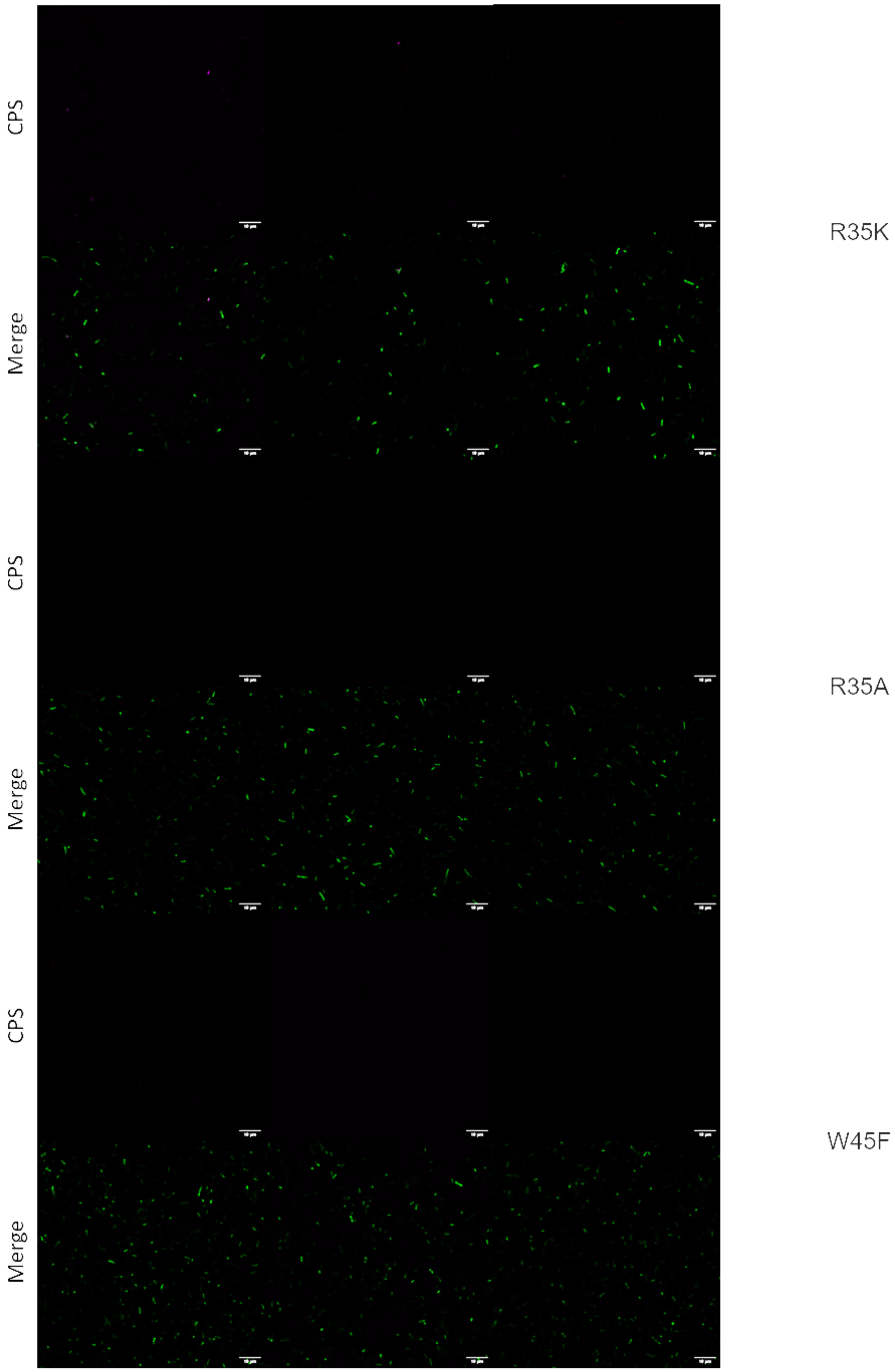

Supplementary Information Figure 24b

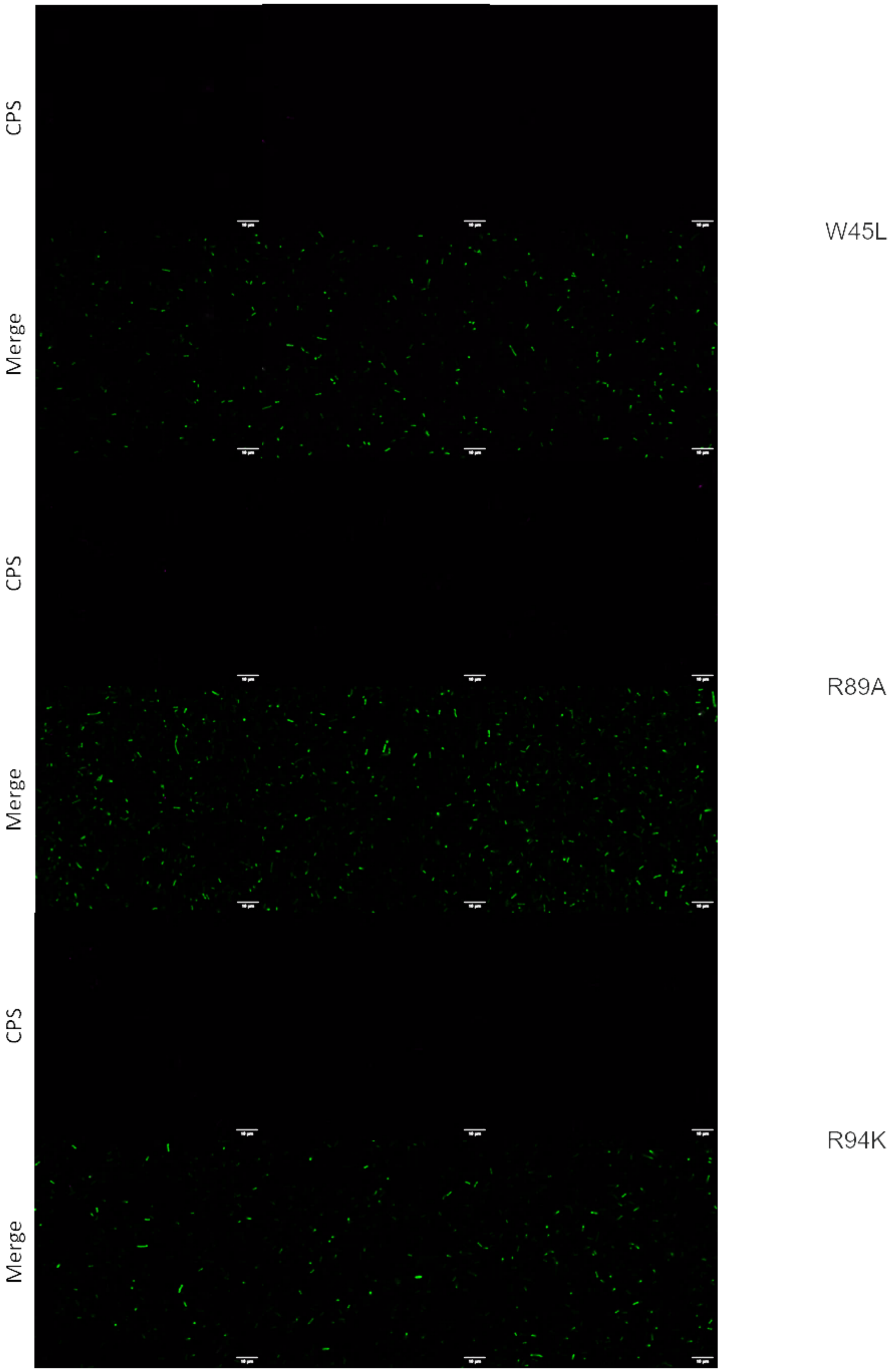

Supplementary Information Figure 24c

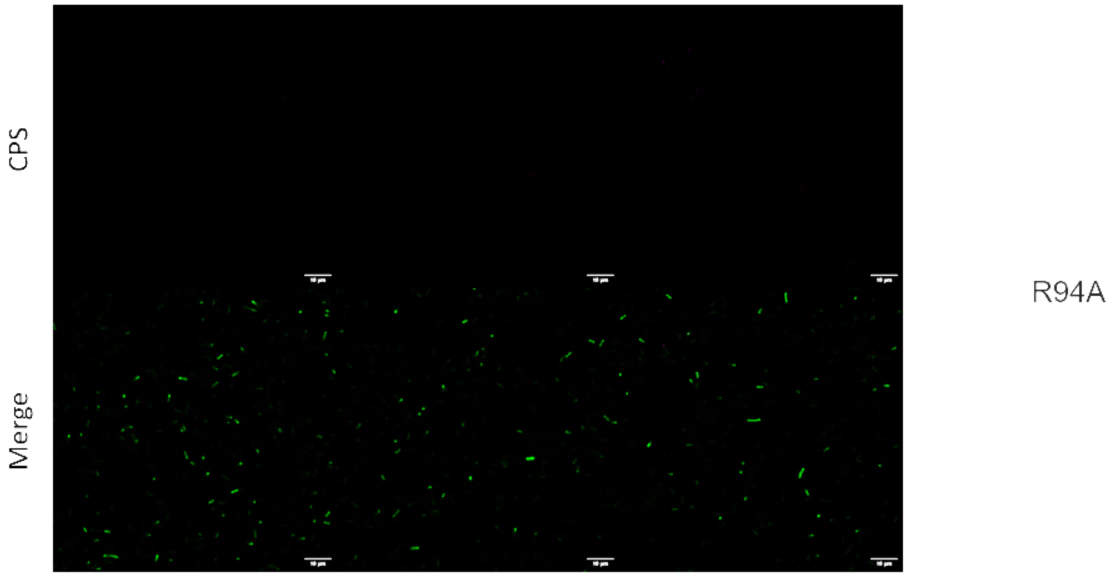

SI Figure 24 (a-c) | Representative full fields of view and additional examples of confocal imaging presented in ED Figure 7. Scalebar: 10µm.

Supplementary Table 1. Chain Completeness

Protein	Chain ID	Apo 1	ATP-bound	ADP:AlF_4^- bound	Apo 2	Glycolipid 1	Glycolipid 2
**KpsT**	A	1-11,17-219	1-216	2-216	Rigid body fit (1-216)	Rigid body fit (1-216)	Rigid body fit (1-216)
	B	1-11,17-219	1-216	2-216	Rigid body fit (1-216)	Rigid body fit (1-216)	Rigid body fit (1-216)
**KpsM**	C	14-268	12-270	14-267	13-269	12-269	14-269
	D	14-268	14-267	14-267	13-269	14-269	14-269
**KpsE**	E	6-50,71-179,320-363	10-50, 71-204,289-368	12-48,73-180,321-361	10-48, 72-176,319-367	10-50,72-178,320-367	7-50, 72-185, 301-370
	F	4-50,72-176,321-371	4-50, 72-206, 289-371	7-48,72-175,322-370	6-48,72-172,321-370	3-52,72-179,319-370	6-50, 72-180, 299-371
	G	5-50,71-180,319-371	5-50, 71-204, 289-371	11-49,71,-175,320-371	10-49,72-177,320-370	9-50,72-181,305-371	6-50, 71-184, 299-370
	H	19-50,71-180,320-351	11-50, 71-204, 290-369	10-48,71-178,320-366	13-48,71-177,315-369	10-50,71-189,299-369	4-50, 71- 192, 299-369
	I	6-50,71-179,320-363	10-50, 70-204, 291-365	12-48,73-180,321-361	10-48,72-176,319-367	10-50,71-188,300-367	9-51, 71-187, 298-369
	J	4-50,72-176,321-371	9-50, 71-204, 289-371	7-48,72-175,322-370	6-48,72-172,321-370	3-50,72-183,308-370	3-50, 71-182, 299-370
	K	5-50,71-180,319-371	13-50, 71-202, 291-371	11-49,71,-175,320-371	10-49,72-177,320-370	6-50,72-189,301-371	6-50, 71-187,300-370
	L	16-50,71-180,320-351	10-50, 71-204, 290-367	10-48,71-178,320-366	13-48,71-177,315-369	10-50,72-183,303-369	4-50, 71-190, 302-371
